# Chiral self-sorting and guest recognition of porous aromatic cages

**DOI:** 10.1038/s41467-022-31785-4

**Published:** 2022-07-11

**Authors:** Dong-Xu Cui, Yun Geng, Jun-Ning Kou, Guo-Gang Shan, Chun-Yi Sun, Kun-Hao Zhang, Xin-Long Wang, Zhong-Min Su

**Affiliations:** 1 Institute of Functional Materials, Department of Chemistry, Northeast Normal University, Changchun, Jilin China; 2 Shanghai Synchrotron Radiation Facility (SSRF), Shanghai Advanced Research Institute, Chinese Academy of Sciences, Shanghai, China; 3 State Key Laboratory of Supramolecular Structure and Materials, Institute of Theoretical Chemistry, College of Chemistry, Jilin University, Changchun, Jilin China

**Keywords:** Self-assembly, Molecular capsules

## Abstract

The synthesis of ultra-stable chiral porous organic cages (POCs) and their controllable chiral self-sorting at the molecular and supramolecular level remains challening. Herein, we report the design and synthesis of a serial of axially chiral porous aromatic cages (PAC **1-*S*** and **1-*R***) with high chemical stability. The theoretical and experimental studies on the chiral self-sorting reveal that the exclusive self-recognition on cage formation is an enthalpy-driven process while the chiral narcissistic and self-sorting on supramolecular assembly of racemic cages can be precisely regulated by π–π and C–H…π interactions from different solvents. Regarding the chemical stability, the crystallinity of PAC **1** is maintained in aqueous solvents, such as boiling water, high-concentrated acid and alkali; mixtures of solvents, such as 1 M H_2_SO_4_/MeOH/H_2_O solution, are also tolerated. Investigations on the chiral sensing performance show that PAC **1** enables enantioselective recognition of axially chiral biaryl molecules.

## Abstract (web summary)

The synthesis of stable chiral porous organic cages and the study of their chiral self-sorting properties is challenging. Here, the authors report axially chiral porous aromatic cages with high stability and solvent-controlled chiral self-sorting.

## Introduction

Chiral self-sorting via covalent and non-covalent bonds is a natural phenomenon, which presents a dominant ability in guiding biologic systems toward the formation of specific products out of equally multiple possibilities^1–4^. The study of external stimuli and intrinsic factors that affect chiral self-sorting in artificial systems could not only provide comprehension of recognition discipline in nature but open new opportunities for the preparation of homochiral materials and related applications for chiral sensing and asymmetric transformation^5^. Controllable processes will be helpful to acquire a deeper understanding of the principles in chiral self-sorting. Compared with covalent and coordination bonds, the difficulty in governing the direction of intermolecular interactions makes it very hard to realize controllable chiral self-sorting. It is well known that weak molecular interactions significantly affect the supramolecular-level assembly in organisms. Therefore, it is of great significance to understand the controllable chiral self-sorting at the supramolecular level. Recently, chiral self-sorting of porous organic cages (POCs) has drawn increasing attention^6,7^. POCs are constructed by dynamic condensation reactions and the discrete molecular cages are held together by intermolecular interactions^8–11^. Such structure feature provides a platform for the study of chiral self-sorting from covalent to weak non-covalent bonds^8,12–15^. Researches on assembling racemic small molecules into cages have been reported and selective chiral self-sorting has been achieved in the covalent cage formation^16–28^. For example, Cao et al. demonstrated chiral self-sorting driven by the intramolecular interactions, which results in [4 + 4] radical homochiral cages^20^. They also prepared the largest [4 + 6] imine cages formed by narcissistic chiral self-sorting^27^. Mastalerz and co-workers reported the chiral self-sorting of giant cubic [8 + 12] salicylimine cages, and only the enantiopure and a meso cage were obtained out of 23 possible cage isomers^7^. This is the largest number of components used so far in social or narcissistic self-sorting. They also presented the selective social self-sorting based on different solubility between homochiral and heterochiral salicylimine cages^16^. In spite of these, the exclusive homochiral self-sorting in different solvent systems remains a challenge^17–23^. More significantly, the supramolecular-level chiral self-sorting remains underexplored.

For the synthesis of chiral POCs, imine condensation has been the prevailing route, and a large number of structures have been reported, normally adopting flexible skeletons with central chiral amines as building blocks^6,10,11,20,29–34^. Although self-correction mechanism of the reversible covalent bond greatly facilitates the accessibility of chiral POCs, the intrinsic dynamic character of imine makes this bond low-energetic stability, and prone to be attacked e.g. by nucleophiles, leading to structural decomposition even in moisture ambient humidity^35–40^. Poor chemical stability has been a thorny issue faced by chiral imine cages and restricts their practical applications^41–43^. Recently, post-synthetic modifications have been proposed as a straightforward way to enhance their stability^34,35,44–47^. For example, Cooper and co-workers converted the imine in CC3 to an amine, and the resulting structure can be stable over a wide pH range (pH = 1.7–12.3)^37^. Mastalerz et al. reported the transformation of imine bonds in [4 + 6] salicylimine cage into chemically robust amide bonds via Pinnick oxidation^45^. Furthermore, they utilized the Povarov reaction and the subsequent oxidation to transform the salicylimine cage into a quinoline cage which is stable under harsh acidic and basic conditions^46^. In addition, they introduced a two-step approach for the synthesis of a carbamate cage from [2 + 3] imine cage and the structure can be preserved in 1 M HCl and NaOH for at least 16 h^47^. We can find these methods either introduce additional flexibility of skeletons, which increases the loss risk of internal pores^36–38^, or require multistep synthesis which raises the synthetic cost and difficulties. For broad applications, it is essential to build highly chemically stable chiral POCs via a facile approach.

Herein, we report the one-pot synthesis of ultra-stable axially chiral porous aromatic cages (PAC **1-*S*** and **1**-***R***) by condensation of binaphthylenediamine (BINAM) and 2,4,6-triformylphloroglucinol (TFP), and their chiral recognition. Structural analysis reveals that PAC **1-*S*** or -***R*** is an [4 + 6] octahedral aromatic cage. As far as we know, this is one of the few examples of chiral POCs with the aromatic structure reported to date. The keto-enol tautomerism-induced conversion of imine bonds to amine is observed in the cage assembly, which, coupled with the hydrophobic environment provided by the aromatic skeleton, makes the cage highly chemical stable under harsh conditions of acid and base. On chiral self-sorting, exclusively narcissistic self-sorting is observed in the formation of the cage while the chiral self-recognition of supramolecular assembly of racemic structure can be controllably adjusted by organic solvent. Moreover, the crystalline homochiral cage and the racemic structure can reversible transformation. Notably, the homochiral structure shows enantioselective recognition toward axially chiral aromatic racemates.

## Results

### Synthesis and structure analysis of chiral enantiomer of PAC 1

Homochiral PAC **1-*S*** and -***R*** were directly synthesized by using commercially available TFP and enantiomerically pure BINAM under toluene solvothermal conditions. The chirality of PAC **1-*S*** and -***R*** is confirmed by circular dichroism (CD) spectra which exhibit nearly perfect mirror images in the range of 230−525 nm (Supplementary Fig. 2). The successful condensation and the purity of the product are demonstrated by ^1^H NMR, ^13^C NMR spectra (Supplementary Figs. 3 and 4), and mass spectrometry (observed m/z = 2330.6, calculated m/z = 2330.551) for **1-*S*** (Supplementary Fig. 5). The disappearance of the ^1^H NMR signal at 12.60 and 12.57 ppm after the addition of D_2_O confirms the presence of an exchangeable NH proton (Supplementary Fig. 6), suggesting the conversion of imine bond to the amine. The keto form was confirmed by ^13^C NMR spectroscopy (Supplementary Fig. 4) in which a clear signal near 184 ppm is detected, corresponding to the carbonyl carbons.

**Fig. 1:**
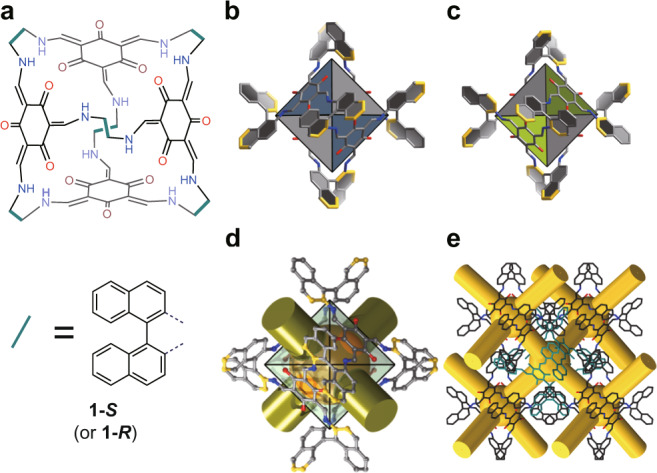
Structure of chiral enantiomer of PAC **1**. **a** Chemical structure of the ***S*** or ***R*** cage in PAC **1**. **b**, **c** The polyhedral structure of ***S*** and ***R*** cage in PAC **1** with conjugated planar in blue or green. **d** The representation of cavity and windows in **1-*R***. **e** The packing of **1-*R*** with 3-D diamondoid pore network.

Single-crystal synchrotron X-ray diffraction (SCSXRD) analysis reveals that PAC **1-*S*** or -***R*** is an [4 + 6] octahedral molecular cage composed of four TFP and six BINAM crystallizing in the *F*4_1_32 chiral space group (Figs. 1a, b and Supplementary Table 1). In the octahedral cage, TFP occupies four faces and is connected with the naphthalene ring through C-N bonds. It is worth noting that one naphthyl of BINAM and TFP are nearly coplanar (dihedral angle: 2.558°). The lengths of the C–N and C–C bond range from 1.365 to 1.384 Å, which are between the lengths of the single (1.47–1.58 Å) and double (1.25–1.34 Å) bond, indicating the formation of delocalized conjugated structures (Supplementary Fig. 7)^48^. Until now, the combination of flexible alicyclic or aliphatic amines with aromatic aldehydes has been a common strategy for the synthesis of imine-based organic cages, especially chiral organic cages. The construction of chiral PACs remains a great challenge since aromatic amines and aldehydes tend to form infinitely extending networks. As far as we know, this is one of the few examples of chiral POCs having the aromatic structure reported to date.

This configuration leads to the dihedral angle of two adjacent planes (73.505°) slightly smaller than the binaphthalene diamine monomer (79.254°). The length of C–O bonds on phloroglucinol changes from 1.43 to 1.249 Å illuminating phenolic hydroxyl functional groups are rearranged from the enol to ketone form^49–51^. This rearrangement makes the aldehyde group more active than ordinary aromatic aldehydes, which facilitates the condensation reaction towards C–N bond formation. The FTIR spectrum (Supplementary Figs. 8 and 9) of **1-*S*** does not show any characteristic stretching bands of imine (C=N) groups, instead, they present obvious C=C stretching ~1602 cm^−1^ and C-N stretching ~1282 cm^−1^. These peaks support the formation of enol–to–keto tautomerism. The cavity of the cage is 13.9 × 13.9 Å^2^. The estimated volume of the internal cavity is *ca*. 229.689 Å^3^, calculating by VOIDOO program with a 2.1 Å probe based on the crystal structure (Supplementary Fig. 10)^52^. These discrete octahedral cages packing in a window-to-window model generate a 3-D diamondoid porous network (Fig. 1d, e).

The porous nature of PAC **1-*S*** and **1-*R*** is probed by N_2_ adsorption at 1.0 bar under 77 K. The adsorption isotherms are type I, suggesting the presence of micropores in the structures, which is consistent with the experimental observations (Supplementary Fig. 11). They have similar total gas uptake of ~205 cm^3^/g, as well as a close apparent BET surface area of ~343 m^2^/g. Pore size distribution shows a narrow range with a pore-width of 13.9 Å (Supplementary Figs. 11 and 12). From the PXRD patterns of **1-*S*** after the N_2_ adsorption experiment, we can find there are no obvious changes in the structure after activation and N_2_ adsorption (Supplementary Fig. 13).

### Chiral self-sorting behavior in cage formation

**Fig. 2:**
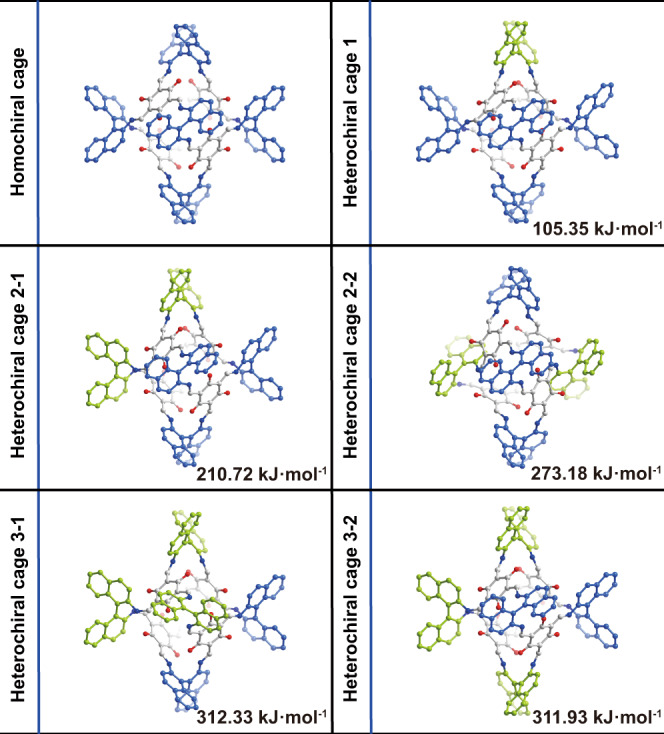
The optimized cage structures based on DFT calculations. The possible cage isomers (enantiomers not included) and their corresponding enthalpies (Δ*H*) kJ·mol^−1^. blue binaphthyl: (S)-enantiomer, yellow binaphthyl: (R)-enantiomer.

**Fig. 3:**
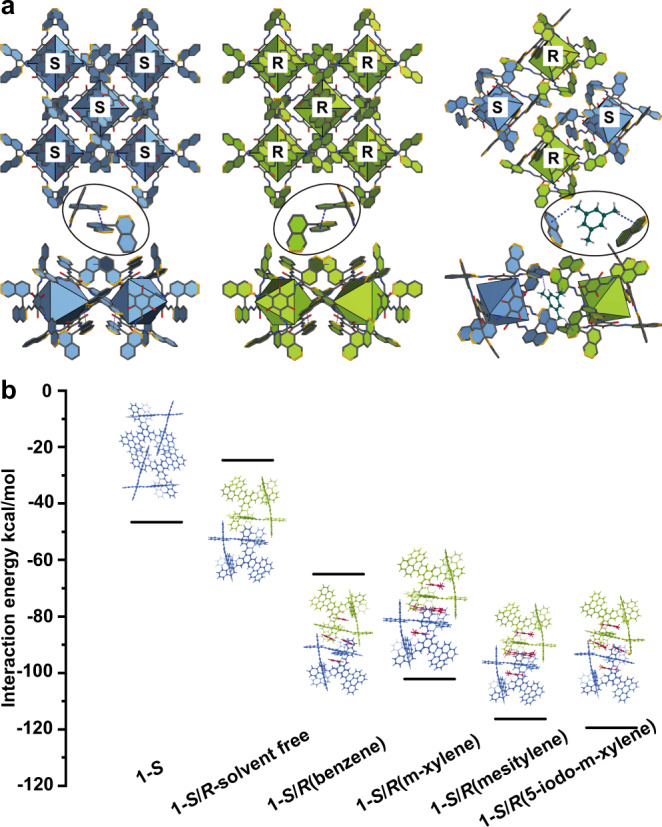
The structures of the cage-cage assembly and interaction energies analysis by DFT calculations. **a** Schematic representation of the interaction between cages and also between solvent molecules and cages in homochiral and heterochiral structures. **b** The interaction energies calculated at PBE0-D3/6-31 G(d,p) level for staking structures.

When using racemic BINAM to react with TFP, the obtained single cage (***S*** and ***R*** cage) is homochirality, as confirmed by ^1^H NMR after reaction using benzene-d6 as solvent and SCSXRD signal (Supplementary Fig. 14 and Fig. 3a). The universality of the exclusive chiral narcissistic self-sorting in single cage formation is also observed when replacing benzene with toluene, mesitylene, p-xylene, m-xylene, 1,2,4-trimethylbenzene, 1,2,3-trimethylbenzene or 5-iodo-m-xylene, as verified by the structures based on SCSXRD and PXRD (Supplementary Figs. 15–17, Supplementary Table 2). These results suggest that the chiral self-sorting on single cage assembly is not disturbed by these solvents, and the homochiral cage may be a thermodynamic product. Such exclusive chiral narcissistic self-sorting is not common in POCs^16,18,29^. According to Burnside’s lemma, 11 cage isomers (including enantiomers) are possible. Eliminating the enantiomers, the possible cages are six (Fig. 2). In dynamic covalent condensation, the major products are the most thermodynamically stable or those with the lowest Gibbs free energy, in which both enthalpy and entropy have a role. To acquire more insights into factors for the favored formation of the homochiral cage, the enthalpy (Δ*H*) and entropy (Δ*S*) at 298.15 K of all possible isomers were calculated by density functional theory (DFT) calculations at PBE0/6-31 G(d,p) level (Supplementary Table 4, Fig. 2). The largest energy difference of Δ*S* between the homochiral and heterochiral structure is only 15.11 J ∙ K^−1^ ∙ mol^−1^ while the energy difference of Δ*H* reaches 150.35-311.94 kJ mol^–1^ and Δ*H* favors the formation of a homochiral cage over the heterochiral structure. This huge energy difference leads to Δ*G* of homochiral cage lower than that of heterochiral ones by 102.25-316.44 kJ mol^−1^. These results illuminate homochiral cage may be the thermodynamically stable conformation and the exclusive chiral narcissistic self-sorting may be an enthalpy-driven process.

### Chiral self-sorting on supramolecular level

**Fig. 4:**
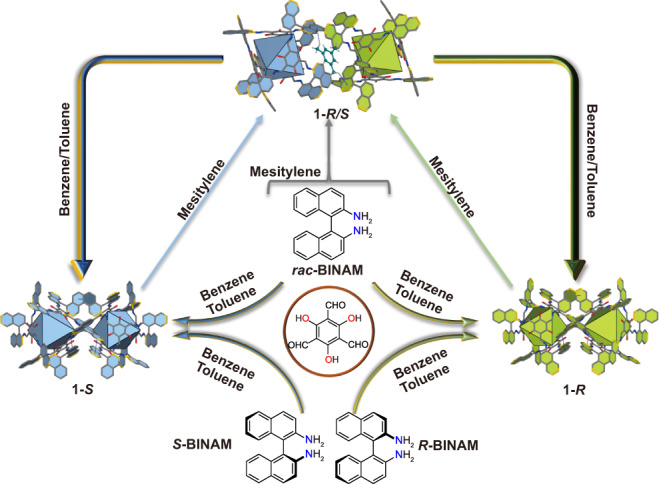
Schematic illustration of structural formation. The synthesis of **1** and the reversible supramolecular structural transformation between homochiral and racemic structures under different solvents.

The supramolecular assembly of ***S*** and ***R*** cages exhibit controllable chiral self-discrimination and self-recognition via precisely adjusting organic solvents. When the solvent is mesitylene, the racemic structure (**1-*R*/*S***) is obtained (Fig. 3a). SCXRD analysis shows **1-*R*/*S*** crystallizes in the *P*-1 space group, where two enantiomer cages are stacked in wall-to-wall manners with the cavities and intermolecular space occupied by mesitylenes (Supplementary Fig. 18). The purity of the sample is illuminated by PXRD (Supplementary Fig. 19). When mesitylene is replaced with toluene, homochiral structure is generated (Fig. 3a). The structure is the same as that prepared from the enantiomerically pure precursor. Interestingly, the homochiral and racemic crystallization presents supramolecular structural reversible transformations (Fig. 4). When equimolar crystals of **1-*S*** and **1-*R*** were dissolved in mesitylene and heated at 120 °C for 3.5 days, the crystals of **1-*R*/*S***⊃**mesitylene** were obtained as confirmed by SCXRD and PXRD (Supplementary Fig. 20). The obtained racemic co-crystals can return to homochiral crystallization in toluene (Supplementary Fig. 20). While it has been reported the transformation of the crystals of homochiral structure to the racemate^16–19^, controllable reversible transformation between a single crystal of homochiral and racemic structure is rare.

DFT calculations based on periodic crystal structures are carried out to clarify the mechanism of solvent-controlled chiral self-sorting. Via reduced density gradient (RDG) analysis, the intuitive information about molecular interactions for homochiral cage and racemic structure are shown in Fig. 3b and S21, S22^53^. The interaction energy of the homochiral cage is −46.6 kcal/mol via strong π–π interactions between adjacent cages. If the neighboring cages are heterochiral, the interaction energy sharply increases to −24.7 kcal/mol and the intermolecular distance is increased in comparison with homochiral configuration. These results demonstrate that the homochiral packing is more stable and preferred than the solvent-free heterochiral structure. Besides, the interaction energy of the solvent (e.g. toluene) included homochiral structure was also explored, although the solvent molecules weren’t determined by X-ray diffraction. In the optimized structure (Fig. S22), the original packing of homochiral cages is kept and three toluene molecules are encapsulated, two of which reside separately inside the cages and one locates between the cages. The interaction energy of this structure is −69.6 kcal/mol which is −3 kcal/mol lower than the corresponding heterochiral structure, suggesting the homochiral structure is preferential even if solvent is included in the configuration. As for the racemic **1-*R*/*S***⊃**mesitylene**, the interaction energy is as low as −116.3 kcal/mol benefiting from the π–π and C–H…π interactions between mesitylenes and cages. Subsequently, mesitylene embedded homochiral structure and the interaction energy is simulated (Fig. S22). The optimized structure contains three mesitylene molecules with two molecules locating in the inner cages and one between the cages. The interaction energy is of −91.8 kcal/mol which is quite higher than that of heterochiral configuration (−116.3 kcal/mol), implying the heterochiral **1-*R*/*S***⊃**mesitylene** is more favorable during chiral self-sorting. It’s worth noting that, mesitylene inclusion leads to the enhanced distance between homochiral cages, implying mesitylene embedded homochiral structure is unrealistic.

In order to comprehensively understand the chiral self-sorting on supramolecular level, a serial of solvent including benzene, p-xylene, m-xylene, 1,2,4-trimethylbenzene, 1,2,3-trimethylbenzene and 5-x-m-xylene (x=Cl, Br and I) are employed. Under the solvents of benzene, p-xylene, m-xylene, 1,2,4-trimethylbenzene and 1,2,3-trimethylbenzene, homochiral structure is produced exclusively (Supplementary Fig. 15), whereas under 5-chloro-m-xylene, 5-bromo-m-xylene and 5-iodo-m-xylene, heterochiral crystals are obtained (Supplementary Fig. 16). As an example, the single-crystal X-ray diffraction (SCXRD) signal of the crystals under 5-iodo-m-xylene was collected. Structural analysis showed that the arrangement of 5-iodo-m-xylene is similar to mesitylene molecules (Fig. S17). From these results, we can find the heterochiral **1**-***R*/*S*** structure could be generated when two methyl groups on benzene locate at an angle of 120° and the left 5-position is occupied by a functional group, otherwise, homochiral stacking is produced. To illuminate these, DFT calculations were performed (Fig. 3 and S22). The optimized structures show that two methyl groups at an angle of 120° can undertake the C–H…π interaction between the neighboring heterochiral cages, and the left methyl or halogen group plays a space-occupying role (Supplementary Fig. 21). The affluent C–H…π and π–π interactions result in significantly decreased interaction energy from −65.0 to −116.3 kcal/mol and therefor promotes the stability of heterochiral structure.

### Stability of PAC 1

**Fig. 5:**
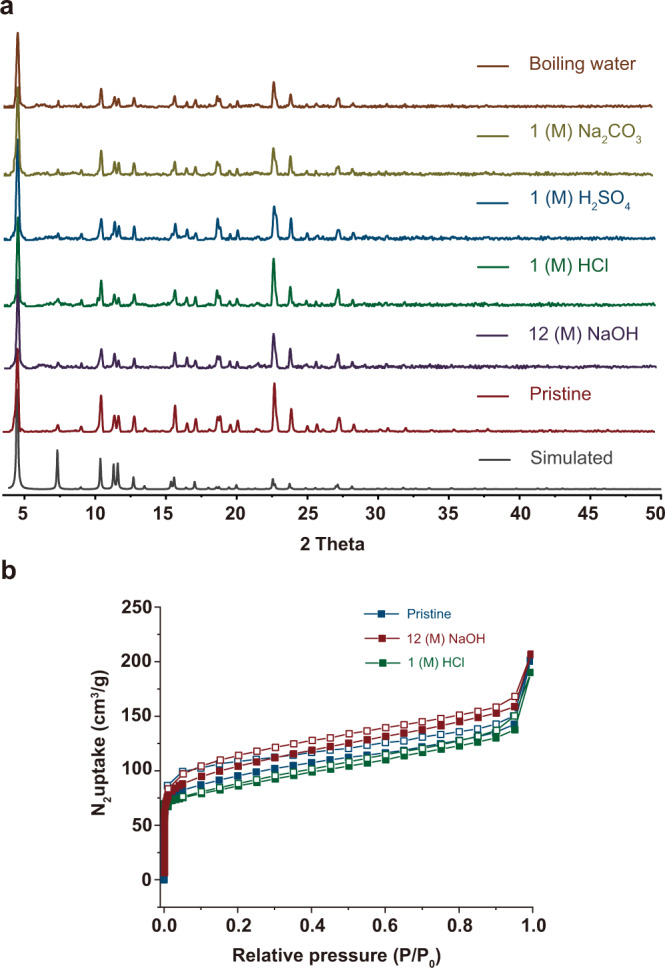
PXRD patterns and N_2_ sorption of **1**-*S* under different harsh conditions. **a** PXRD patterns measured after 7-day treatment of **1-*S*** in boiling water, 1 M H_2_SO_4_, 1 M HCl, 12 M NaOH, and 1 M Na_2_CO_3_. (N_2_ sorption of activated **1-*S*** and **1-*S*** after treatment by NaOH or HCl.

Chemical stability is one of the knotty issues that plague the development of chiral POCs. The chemical stability of homochiral PAC **1-*S*** is investigated under various harsh conditions including boiling water, and concentrated base and acid (12 M NaOH, 1 M HCl and H_2_SO_4_). Excitingly, PAC **1-*S*** presents appealing stability toward the water and also to both concentrated acidic and basic aqueous solution. As confirmed by FTIR and ^1^H NMR (Supplementary Figs. 23 and 24), there is not any chemical decomposition of **1-*S*** when it was soaked in boiling water, NaOH (12 M), HCl (1 M) and H_2_SO_4_ (1 M) aqueous solution for 7 days. From the intact powder X-ray diffraction (PXRD, Fig. 5a) pattern and the crystal photographs (Supplementary Fig. 25), we can find there is no loss of crystallinity, as well. Likewise, these treatments of concentrated acidic and basic aqueous solution do not affect the porous structure in **1-*S*** obviously, as witnessed by the nearly unchanged N_2_ adsorption isotherms after treatment (Fig. 5b). The chemical stability of this PAC towards both alkaline and acid aqueous solution outperforms the reported POCs (Supplementary Table 5). Banerjee et al. reported amine-linked POCs utilizing the phenomena of keto-enol tautomerism and the cages can maintain their structure in hot water, 0.5 M HCl and 1 M NaOH solution^49^. Nevertheless, the keto-enamine structure still faces the risk of instability in alkali, because the second N protonation would lead to the back-conversion of the keto form to the enol^51^.

To illuminate the key factor of PAC **1-*S*** toward stability under harsh conditions, dedicated experiments were carried out. Firstly, it was observed that in 12 M NaOH or 1 M H_2_SO_4_ aqueous solutions, the sample floated above the aqueous surface after stirring, whereas in 12 M NaOH or 1 M H_2_SO_4_ H_2_O/MeOH solution, the crystals were completely submerged (Supplementary Fig. 26). The high hydrophobicity of the crystal surface under aqueous solution is verified by the water contact angle of 145.7° (Supplementary Fig. 27). Based on these, it is speculated that the robust stability of **1-*S*** in strong acid and alkali aqueous solution mainly originates from the hydrophobicity of the aromatic skeleton.

Following, the sample was wetted by methanol and then immersed in the solutions including water, Na_2_CO_3_, NaOH, HCl and H_2_SO_4_. The PXRD patterns of the sample in water and Na_2_CO_3_ shows little change compared to those of fresh samples (Supplementary Fig. 28), suggesting the stability of the cage in these conditions arises from the irreversible nature of the enol-to-keto tautomerism. PXRD patterns of the sample soaked in 1 M HCl and H_2_SO_4_ (Supplementary Fig. 28) exhibited nearly unchanged patterns. Under more infiltrative environment of 1 M H_2_SO_4_/MeOH/H_2_O solution, the sample still has a flawless crystalline appearance (Supplementary Fig. 29) and the SCSXRD signals show that its structure is similar to the one before soaking (Supplementary Table 6). On the basis of these experiments, it seems that the disappearance of the acid-labile imine (C=N) bond rather than the hydrophobic effect dominate the stability in this condition. Several new peaks emerge in the PXRD patterns of the sample in 12 M NaOH (Supplementary Fig. 28). When immersed the crystal in 12 M NaOH/MeOH/H_2_O solution, its quality became poor. To understand the changes on the sample, scanning electron microscopy (SEM) were employed. Via scanning the cross-section of a crystal, we can find a certain amount of Na ions are present (Supplementary Fig. 30). These results demonstrate that hydrophobic characteristics play an important role in protecting the cage from hydrolysis in concentrated bases conditions. In a word, the keto-enamine structure of organic cages may play an important role in the stabilization in acidic media while the hydrophobicity is crucial for alkali resistance.

In thermal stability, **1-*S*** also has attractive performance. Through thermogravimetric analysis (Supplementary Fig. 31), it is found there is a weight loss of about 8% before 90 °C, which can be attributed to the loss of solvent molecules. Then, a platform appears before 200 °C, indicating the structure can be stabilized to this temperature. To further prove its thermal stability, a variable temperature PXRD test was carried out with an interval of 50 °C (Supplementary Fig. 32). No obvious change is observed, suggesting **1-*S*** can be stable before 200 °C.

### Enantioselective recognition of biaryl molecules

**Fig. 6:**
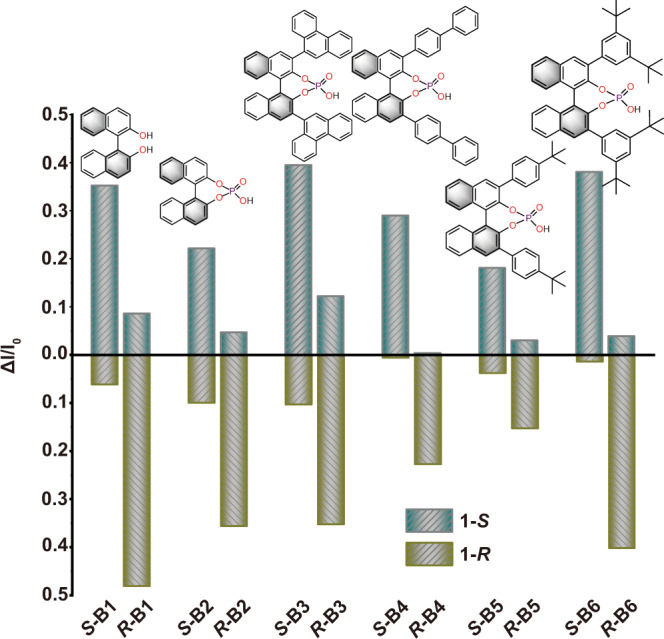
Ratio of luminescent intensity. Ratio of luminescent intensity after adding **1-*S*** (dark blue column) and **1-*R*** (brown column) into the solution of axial chiral enantiomers (**B1**-**B6**).

As crucial components in the biological system, the enantiomer recognition of atropisomerically biaryl molecules is of significant importance to life sciences and pharmaceutics^54,55^. The observed supramolecular interactions may endow PAC **1** capacity for enantioselective recognition of biaryl molecules^56^. Therefore, homochiral **1-*S*** and **1-*R*** is selected as a sensor for six enantiomers **B1-B6**. Chiral sensing experiments were conducted by immersing activated enantiopure  PAC **1** into the solution of analytes, separately and evaluated by the fluorescence of analytes. To get a clear evaluation, the changes (ΔI) in luminescence intensity before and after adding PAC **1** are normalized (ΔI/I_0_). As shown in Figs. 6, S33, **1-*S*** preferred *S*-fashion substrates over the *R*-fashion, especially to **B4** with the selectivity up to 98%. Recycle experiments showed the sensing ability maintained by ~85% after five rounds of recycling. (Supplementary Fig. 34). The limit of detection (LOD) of **1-*S*** to ***S*-B4** was determined to be 0.597 *μ*M (Supplementary Figs 35 and 36). A similar selectivity also occurred in **1-*R*** to *R*-fashion substrates. These results demonstrate PAC **1** could be a promising probe for recognition of enantiomer atropisomerically biaryl molecules. Moreover, to clarify the internal reasons for the recognition process, molecular dynamics (MD) simulations and DFT calculations on **1-*S*** to ***R*-B4** and ***S*-B4** were carried out, as detailed in the supporting information. The energy versus simulation time in NPT (Normal Pressure and Temperature) process for **1-*S***/***R***-**B4** and **1-*S***/***S***-**B4** blends suggest good energy convergence (Supplementary Fig. 37). The nearest-neighbor **1-*S***/***R*-B4** and **1-*S***/***S*-B4** dimers were selected from the MD results and then further geometrical optimization was performed at PBE0-D3/6-31 G(d,p) level (Supplementary Figs 38 and 39). The final structures show both ***S*-B4** and ***R*-B4** have close stacking with **1**-***S***. For **1**-***S*/*S*-B4**, the two molecules stack in back-to-back form, while for **1-*S*/*R*-B4**, the two molecules show cross stacking. The calculated interaction energies shown in Supplementary Table 7 indicate they have similar stability. Then the excitation energy transfer between **1-*S*** and ***S*-B4** (***R*-B4**) was estimated based on the above stable structures. It is found that the excitation energy transfer of ***1-S***/***S-B4*** has a larger electronic coupling than that of **1*-S***/***R*-B4**, suggesting more efficient energy transfer between **1-*S*** and ***S*-B4**. This may result in the observed larger changes (Δ*I*) in luminescence intensity.

## Discussion

In conclusion, a pair of axially chiral [4 + 6] PAC (**1-*S*** and **1-*R***) is constructed from the condensation of binaphthylenediamine and tridentate aldehyde. As far as we know, this is one of the few examples of chiral POCs having the aromatic structure reported to date. The keto-enol tautomerism-induced conversion of imine to amine is observed in the cage assembly. The keto-enamine structure renders PAC **1** an exceptional stability under water and concentrated acid while the hydrophobic characteristic is crucial for alkali resistance. Controllable chiral self-sorting at both molecular and supramolecular levels is observed. DFT calculations show that the exclusive self-recognition on single cage formation is an enthalpy-driven process, while the narcissistic and social self-sorting on cage-cage supramolecular assembly is regulated by different solvents via C–H…π and π–π interactions. The reversible transformation of the homochiral cage and racemic structure is studied by single-crystal X-ray diffraction. Moreover, enantiopure PAC **1** possesses good enantioselectivity toward a series of axially chiral aromatic racemates. This work provides insights for the design of chiral porous aromatic cages and the related chiral self-sorting, and these robust chiral POCs may offer new prospects for the application of organic cages.

## Methods

### Synthesis of 1-*S* and 1-*R*

A mixture of *S*-BINAM ([*S*]-[–]-1,1′-Binaphthyl-2,2′-diamine) or *R*-BINAM ([*R*]-[ + ]-1,1′-Binaphthyl-2,2′-diamine) (0.1 mmol) and 2,4,6-Triformylphloroglucinol (0.066 mmol) was dissolved in CH_3_(CH_2_)_3_OH (1 mL), toluene (5 mL) and acetic acid 6 M (0.6 mL). After stirring for 10 min at room temperature, this solution was transferred into a Parr Teflon–lined autoclave and kept at 120 °C for 72 h. After cooling down to room temperature, red octahedral crystals were obtained. Yield: 42 % based on 2,4,6-Triformylphloroglucinol. Elemental analysis Calcd: C, 80.41; H, 4.12; N, 7.22. Found: C, 80.24; H, 4.30; N, 6.98.

### Synthesis of 1-*R*/*S*⊃mesitylene

A mixture of *S*-BINAM and *R*-BINAM (1,1′-Binaphthyl-2,2′-diamine) (0.1 mmol) and 2,4,6-Triformylphloroglucinol (0.066 mmol) was dissolved in acetic acid 6 M (0.6 mL), CH_3_(CH_2_)_3_OH (1 mL) and mesitylene (5 mL). After stirring for 10 min, this solution was transferred into a Parr Teflon–lined autoclave and kept at 120 °C for 72 h. After cooling down to room temperature, red rod crystals were obtained and washed with CH_3_OH. Yield: 48 % based on 2,4,6-Triformylphloroglucinol. Elemental analysis Calcd: C, 82.05; H, 5.13; N, 5.98. Found: C, 81.84; H, 5.36; N, 5.77.

### Synthesis of 1-*R*/*S*⊃5-iodo-m-xylene

Single crystals of **1-*R*/*S***⊃**5-iodo-m-xylene** were synthesized under similar conditions apart from the solvent which was replaced with 5-iodo-m-xylene. After slow cooling to room temperature, red rod crystals were obtained with a yield of 38 % based on 2,4,6-Triformylphloroglucinol. Elemental analysis Calcd: C, 69.54; H, 4.07; N, 5.18. Found: C, 69.81; H, 4.22; N, 4.96.

### Structural transformation from 1-*S* and 1-*R* to 1-*R*/*S*⊃mesitylene

The crystalline samples of **1-*S*** (0.01 g) and **1-*R*** (0.01 g) were put in acetic acid 6 M (aq) (0.6 mL), CH_3_(CH_2_)_3_OH (1 mL) and mesitylene (5 mL) in a Parr Teflon-lined stainless steel vessel and heated at 120 °C for 72 h. After cooling to room temperature, red rod crystals **1-*R*/*S***⊃**mesitylene** were obtained. Single crystal X-ray diffraction confirmed that the structure of **1-*S*** and **1-*R*** was transformed into **1-*R*/*S***⊃**mesitylene**.

### Structural transformation from 1-*R*/*S*⊃mesitylene to 1-*S* and 1-*R*

The crystalline samples of **1-*R*/*S***⊃**mesitylene** (0.02 g) were put in acetic acid 6 M (aq) (0.6 mL), CH_3_(CH_2_)_3_OH (1 mL) and toluene (5 mL) in a Parr Teflon-lined stainless steel vessel and heated at 120 °C for 72 h. After cooling to room temperature, red octahedral crystals **1-*S*** and **1-*R*** were obtained. Single crystal X-ray diffraction confirmed that the structure of **1-*R*/*S***⊃**mesitylene** was transformed into **1-*S*** and **1-*R***.

### Chemical stability

Cage **1** (10 mg) was soaked in 12 M NaOH, 1 M Na_2_CO_3_, 1 M HCl and H_2_SO_4_, and boiling water for 7 days. After that, the soaked crystals were filtered and washed with water (10 mL×5), ethanol (10 mL) and ether (20 mL). After vacuum drying, these samples were used for various tests, including IR, PXRD, elemental analysis, ^1^H NMR and N_2_ adsorption. For N_2_ adsorption, the sample (100 mg) needs to be further soaked in ether (5 mL×6, 12 h) and vacuum activation.

### Chemical sensing

The crystal (5 mg) used for detection was placed in the standard solution (EtOH/H_2_O, 1 mL/1 mL) of the analyte. The fluorescence spectra were recorded after 30 minutes under the excitation wavelength of 370 nm. For the recycling experiment, the samples used in the first round of experiments were filtered out and then soaked in fresh ethanol solution. Replace with fresh ethanol solvent every 15 minutes and repeat several times. The regenerated sample is placed in a fresh solution of the analyte (B4) for 30 minutes, and then the fluorescence intensity is collected. Repeat the above steps 5 times.

## Supplementary information

Supplementary Information

## Data Availability

The crystallographic data generated in this study have been deposited in the database of Cambridge Crystallographic Data Centre (https://ccdc.cam.ac.uk) under accession code: CCDC number 2036617 for **1-*****R***, 2036618 for **1-*****S***, 2036619 for **1-*****R*****/*****S***⊃**5-iodo-m-xylene** and 2036620 for **1-*****R*****/*****S***⊃**mesitylene**. The data generated in this study are provided in the manuscript, its Supplementary Information, or can be obtained from the authors upon request. The xyz coordinates for the simulated structures can be found as source data. Source data are provided with this paper.

## Source data

Source Data

## References

[1] Wu A, Isaacs L (2003). Self-Sorting:  The Exception or the Rule?. J. Am. Chem. Soc..

[2] Jędrzejewska H, Szumna A (2017). Making a Right or Left Choice: Chiral Self-Sorting as a Tool for the Formation of Discrete Complex Structures. Chem. Rev..

[3] Liu M, Zhang L, Wang T (2015). Supramolecular Chirality in Self-Assembled Systems. Chem. Rev..

[4] Dong J, Liu Y, Cui Y (2021). Supramolecular Chirality in Metal-Organic Complexes. Acc. Chem. Res.

[5] Li C, Zuo Y, Zhao Y-Q, Zhang S (2020). Chiral Self-sorting in Cage-like Compounds. Chem. Lett..

[6] Acharyya K, Mukherjee PS (2019). Organic Imine Cages: Molecular Marriage and Applications. Angew. Chem. Int. Ed..

[7] Wagner P (2021). Chiral Self-sorting of Giant Cubic [8+12] Salicylimine Cage Compounds. Angew. Chem. Int. Ed..

[8] Sun Y, Chen C, Liu J, Stang PJ (2020). Recent Developments in the Construction and Applications of Platinum-based Metallacycles and Metallacages via Coordination. Chem. Soc. Rev..

[9] Rowan SJ, Cantrill SJ, Cousins GRL, Sanders JKM, Stoddart JF (2002). Dynamic Covalent Chemistry. Angew. Chem. Int. Ed..

[10] Hasell T, Cooper AI (2016). Porous Organic Cages: Soluble, Modular and Molecular Pores. Nat. Rev. Mater..

[11] Mastalerz M (2018). Porous Shape-Persistent Organic Cage Compounds of Different Size, Geometry, and Function. Acc. Chem. Res..

[12] Gong Y (2019). Bottom-Up Construction and Reversible Structural Transformation of Supramolecular Isomers based on Large Truncated Tetrahedra. Angew. Chem. Int. Ed..

[13] Ye Y (2015). Self-Assembly of Chiral Metallacycles and Metallacages from a Directionally Adaptable BINOL-Derived Donor. J. Am. Chem. Soc..

[14] Zhang J (2018). Asymmetric Phosphoric Acid-catalyzed Four-component Ugi Reaction. Science.

[15] Zhu H (2020). Formation of Planar Chiral Platinum Triangles via Pillar[5]arene for Circularly Polarized Luminescence. J. Am. Chem. Soc..

[16] Beaudoin D, Rominger F, Mastalerz M (2017). Chiral Self-Sorting of [2+3] Salicylimine Cage Compounds. Angew. Chem. Int. Ed..

[17] Sisco SW, Moore JS (2014). Homochiral Self-Sorting of BINOL Macrocycles. Chem. Sci..

[18] Schafer LL, Tilley TD (2001). Efficient Diastereoselective Syntheses of Chiral Macrocycles via Zirconocene Coupling. Synthetic Control of Size and Geometry. J. Am. Chem. Soc..

[19] Jones JTA (2011). Modular and Predictable Assembly of Porous Organic Molecular Crystals. Nature.

[20] Jiao T, Qu H, Tong L, Cao X, Li H (2021). A Self-Assembled Homochiral Radical Cage with Paramagnetic Behaviors. Angew. Chem. Int. Ed..

[21] Wang X (2016). Assembled Molecular Face-rotating Polyhedra to Transfer Chirality from Two to Three Dimensions. Nat. Commun..

[22] Qu H (2020). Truncated Face-Rotating Polyhedra Constructed from Pentagonal Pentaphenylpyrrole through Graph Theory. J. Am. Chem. Soc..

[23] Qu H (2017). Molecular Face-Rotating Cube with Emergent Chiral and Fluorescence Properties. J. Am. Chem. Soc..

[24] Wang Y (2017). Interconversion of Molecular Face-rotating Polyhedra through Turning inside out. Chem. Commun..

[25] Zhang P (2018). Chiral Separation and Characterization of Triazatruxene-based Face-rotating Polyhedra: the Role of Non-covalent Facial Interactions. Chem. Commun..

[26] Qu H (2018). Chiral Molecular Face-rotating Sandwich Structures Constructed through Restricting the Phenyl Flipping of Tetraphenylethylene. Chem. Sci..

[27] Wang X (2018). Narcissistic Chiral Self-sorting of Molecular Face-rotating Polyhedra. Org. Biol. Chem..

[28] Slater AG (2018). Dissymmetric Porous Organic Cage. Mol. Syst. Des. Eng..

[29] Greenaway RL, Jelfs KE (2020). High-Throughput Approaches for the Discovery of Supramolecular Organic Cages. ChemPlusChem.

[30] Tozawa T (2009). Porous Organic Cages. Nat. Mater..

[31] Hasell T, Chong SY, Jelfs KE, Adams DJ, Cooper AI (2012). Porous Organic Cage Nanocrystals by Solution Mixing. J. Am. Chem. Soc..

[32] Su K (2020). Reticular Chemistry in the Construction of Porous Organic Cages. J. Am. Chem. Soc..

[33] Zhang L (2020). Desymmetrized Vertex Design toward a Molecular Cage with Unusual Topology. Angew. Chem. Int. Ed..

[34] Wang Y (2018). Elucidation of the Origin of Chiral Amplification in Discrete Molecular Polyhedra. Nat. Commun..

[35] Bera S (2019). Porosity Switching in Polymorphic Porous Organic Cages with Exceptional Chemical Stability. Angew. Chem. Int. Ed..

[36] Xu H, Gao J, Jiang D (2015). Stable, Crystalline, Porous, Covalent Organic Frameworks as a Platform for Chiral Organocatalysts. Nat. Chem..

[37] Liu M (2014). Acid- and Base-Stable Porous Organic Cages: Shape Persistence and pH Stability via Post-synthetic “Tying” of a Flexible Amine Cage. J. Am. Chem. Soc..

[38] Swamy SI (2010). A Metal−Organic Framework with a Covalently Prefabricated Porous Organic Linker. J. Am. Chem. Soc..

[39] Turcani L, Greenaway RL, Jelfs KE (2019). Machine Learning for Organic Cage Property Prediction. Chem. Mater..

[40] Jelfs KE (2011). Large Self-Assembled Chiral Organic Cages: Synthesis, Structure, and Shape Persistence. Angew. Chem. Int. Ed..

[41] Lee J-SM, Cooper AI (2020). Advances in Conjugated Microporous Polymers. Chem. Rev..

[42] Yuan Y, Zhu G (2019). Porous Aromatic Frameworks as a Platform for Multifunctional Applications. ACS Cent. Sci..

[43] Yao Z (2015). Dithienopicenocarbazole as the Kernel Module of Low-energy-gap Organic Dyes for Efficient Conversion of Sunlight to Electricity. Energy Environ. Sci..

[44] Schick THG, Lauer JC, Rominger F, Mastalerz M (2019). Transformation of Imine Cages into Hydrocarbon Cages. Angew. Chem..

[45] Bhat AS (2019). Transformation of a [4+6] Salicylbisimine Cage to Chemically Robust Amide Cages. Angew. Chem..

[46] Alexandre PE (2020). A Robust Porous Quinoline Cage: Transformation of a [4+6] Salicylimine Cage by Povarov Cyclization. Angew. Chem. Int. Ed..

[47] Hu XY (2017). Transforming a Chemically Labile [2+3] Imine Cage into a Robust Carbamate Cage. Chem. Commun..

[48] Allen FH (1987). Tables of Bond Lengths Determined by X-ray and Neutron Diffraction. Part 1. Bond Lengths in Organic Compounds. J. Chem. Soc., Perkin Trans..

[49] Bera S (2017). Odd–Even Alternation in Tautomeric Porous Organic Cages with Exceptional Chemical Stability. Angew. Chem. Int. Ed..

[50] Kieryk P, Janczak J, Panek J, Miklitz M, Lisowski J (2016). Chiral 2 + 3 Keto-Enamine Pseudocyclophanes Derived from 1,3,5-Triformylphloroglucinol. Org. Lett..

[51] Kandambeth S (2012). Construction of Crystalline 2D Covalent Organic Frameworks with Remarkable Chemical (Acid/Base) Stability via a Combined Reversible and Irreversible Route. J. Am. Chem. Soc..

[52] Kleywegt GJ, Jones TA (1994). Detection, Delineation, Measurement and Display of Cavities in Macromolecular Structures. Acta Crystallogr D..

[53] Lefebvre C (2017). Accurately Extracting the Signature of Intermolecular Interactions Present in the NCI plot of the Reduced Density Gradient versus Electron Density. Phys. Chem. Chem. Phys..

[54] Bringmann G (2005). Atroposelective Synthesis of Axially Chiral Biaryl Compounds. Angew. Chem. Int. Ed..

[55] Hong T (2020). Chiral Metallacycles as Catalysts for Asymmetric Conjugate Addition of Styrylboronic Acids to α,β-Enones. J. Am. Chem. Soc..

[56] Hou Y (2020). Highly Emissive Perylene Diimide-Based Metallacages and Their Host-Guest Chemistry for Information Encryption. J. Am. Chem. Soc..

