# Supplementary material for: Chiral self-sorting and guest recognition of porous aromatic cages
Source: Nat Commun. 2022 Jul 11;13:4011. doi: 10.1038/s41467-022-31785-4 (PMC9273608; doi:10.1038/s41467-022-31785-4)
Supplement: Supplementary file 1 — Supplementary Information [file 41467_2022_31785_MOESM1_ESM.pdf]

Supporting Information

Chiral Self-sorting and Guest Recognition of Porous Aromatic Cages

Dong-Xu Cui,[1,4] Yun Geng,[1,4] Jun-Ning Kou,[1] Guo-Gang Shan,[1] Chun-Yi Sun,[1] ✉ Kun-Hao Zhang,[2] Xin-Long Wang[1] ✉ & Zhong-Min Su[1,3]

[1]Institute of Functional Materials Chemistry, Northeast Normal University, Changchun, Jilin, China. [2]Shanghai Synchrotron Radiation Facility (SSRF), Shanghai Advanced Research Institute, Chinese Academy of Sciences, Shanghai, China. [3]State Key Laboratory of Supramolecular Structure and Materials, Institute of Theoretical Chemistry, College of Chemistry, Jilin University, Changchun, Jilin, China. [4]These authors contributed equally: Dong-Xu Cui, Yun Geng. ✉e-mail: suncy009@nenu.edu.cn; wangxl824@nenu.edu.cn.

Supplementary Methods

General information. All reagents and solvents were obtained from commercial sources and used without further purification. PXRD patterns were recorded ranging from 5° to 35° at room temperature on a Siemens D5005 diffractometer with Cu K*α* (λ = 1.5418 Å). Elemental analyses (H, N and C) were performed on a Perkin-Elmer 2400 CHN elemental analyzer. The water contact angle (CA, 2 μL) on the samples were examined using a DSA-25; KRÜSS GMBH, Germany instrument at room temperature. Fluorescence of analytes was conducted on anFL-4600 FL spectrophotometer. The scanning electron microscopy images (SEM) were clooected on XL-30 ESEM-FEG Scanning Electron Microscope. The circular dichroism (CD) spectra were measured on a MOS-500 spectrophotometer (KBr pellets). The mass spectrum was measured on a Bruker Daltonics flexAnalysis. ^{1}H NMR spectra were obtained by a Bruker Avance NEO 500 MHz spectrometer. The thermogravimetric analysis (TGA) was performed on a Shimadzu DTG-60H instrument over the temperature range of 20 to 800°C under a nitrogen atmosphere with a heating rate of 10°C/min. Fourier transform infrared (FT-IR) spectra (KBr pellets) were recorded in the range 4000–400 cm^{-1} on an IFS-66V/S using the KBr pellet method. Infrared (IR) spectra were carried out on a Nicolet 6700 Flex FTIR spectrometer equipped with smart iTR™ attenuated total reflectance (ATR) sampling accessory in the range of 550-4000 cm^{-1}.

X–ray Crystallography Studies

The crystallographic data of **1-*R*** and **1-*S*** were performed on the Shanghai Synchrotron Radiation Facility, instrument BL17B. The data frames were collected using the program APEX 4 and processed using the program SAINT routine in APEX 4. Other data collections were performed on a Bruker D8-Venture diffractometer with a Turbo X-ray Source (Cu K*α* radiation, λ = 1.5418 Å) adopting the direct drive rotating anode technique and a CMOS detector at 173 K. The data frames were collected using the program APEX 3 and processed using the program SAINT routine in APEX 3. The structures were solved by direct methods and refined by the full1matrix least-squares on F^2 using the SHELXL-2014 program. The hydrogen atoms of the organic ligand were generated geometrically (C-H = 0.96 Å). The restrained DFIX, SIMU, SADI, RIGU, ISOR instructions were used to make the structures more reasonable. The diffraction intensity of **1-*R*/*S*** is so weak that no diffraction spots can be collected with a resolution higher than 1.2, regardless of Mo or Cu radiation source used. CCDC number of 2036617 for **1-*R***, 2036618 for **1-*S***, 2036619 for **1-*R*/*S*⊃5-iodo-m-xylene** and 2036620 for **1-*R*/*S*⊃mesitylene**.

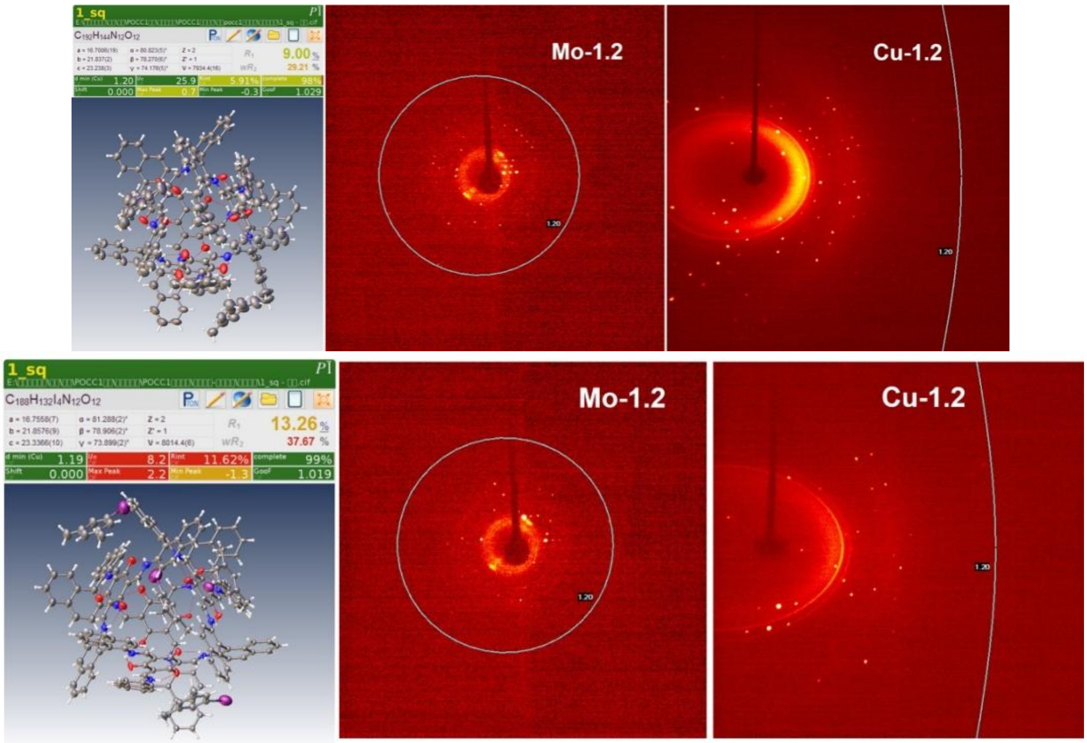

Supplementary Figure 1. Explanation of crystallographic alerts. Explanation of crystallographic alerts of **1-*R/S*⊃mesitylene** (top) and **1-*R/S*⊃5-iodo-m-xylene** (bottom).

Synthetic procedure under different solvents

A mixture of *S*-BINAM and *R*-BINAM (1,1′-Binaphthyl-2,2′-diamine) (0.1 mmol) and 2,4,6-Triformylphloroglucinol (0.066 mmol) was dissolved in acetic acid 6M (0.6 mL), $CH_3(CH_2)_3OH$ (1 mL) and toluene (5 mL). After stirring for 10 min, this solution was transferred into a Parr Teflon–lined autoclave and kept at 120 °C for 72 h.

For other systems, the conditions also keep the same, except replacing toluene with mesitylene, p-xylene, m-xylene, 1,2,4-trimethylbenzene, 1,2,3-trimethylbenzene, 5-chloro-m-xylene, 5-bromo-m-xylene or 5-iodo-m-xylene.

N_2 adsorption

The surface areas and porosities of the cages were characterized by N_2 adsorption and desorption analysis at 77 K using an autosorb computer-controlled surface analyzer on AUTOSORB-iQ2 adsorptometer (Quantachrome Instrument). The Brunauer-Emmett-Teller (BET) surface areas were calculated assuming a value of 0.162 nm^2 for the cross-sectional area of the N_2 molecules. The density function theory (N_2 at 77K on carbon, QSDFT equilibrium model) and isotherm data were used to calculate the pore size distribution.
Activation of the cage compound for gas sorption: 100 mg of the cage compound (crystalline) were soaked in ether (5 mL) with shaking, and the solvent was replaced by fresh ether every 6 hours. After 2 days, the solid was filtered and then dried under a high vacuum (10-2 bar) at 120 °C for 12 h.
Before gas sorption analysis, the sample was degassed at 100 °C for 10 h.

Chemical stability

Cage **1** (10 mg) was put separately in 12 M NaOH (5 mL), 1 M Na_2CO_3 (5 mL), 1 M HCl (5 mL) and H_2SO_4 (5 mL), and boiling water (5 mL) with shaking for 7 days. After that, the crystals were filtered and washed with water (10 mL×5), ethanol (10 mL) and ether (20 mL) in sequence. After vacuum drying at 100 °C for 12 h, these samples were used for various tests, including IR, PXRD, elemental analysis, ^1H NMR and N_2 adsorption.
For N_2 adsorption, the sample (100 mg) after being put in the above harsh condition was filtered and washed with water (10 mL×5), ethanol (10 mL) and ether (20 mL) in sequence. Then, it needs to be further soaked in ether (5 mL) with shaking, and the solvent was replaced by fresh ether every 6 hours. After 2 days, the solid was filtered and then dried under a high vacuum (10-2 bar) at 120 °C for 12 h. Before gas sorption analysis, the sample was degassed at 100 °C for 10 h.
Cage **1** (10 mg) was soaked separately in 12 M NaOH/MeOH/H_2O (MeOH/H_2O=1:1, 5 ml), 1M H_2SO_4/MeOH/H_2O (MeOH/H_2O=1:1, 5 mL) with shaking for 7 days. After that, the crystals were filtered and washed with water and ether for messurements.

Chemical sensing

The crystal (5 mg) used for detection was placed in the standard solution (EtOH/H_2O, 1 mL/1 mL) of the analyte. The fluorescence spectra were recorded after 30 minutes under the excitation wavelength of 370 nm.

Recycling experiment: the samples used in the first round of experiments were filtered out, and then soaked in fresh ethanol solution (2 mL). Replace with fresh ethanol solvent every 15 minutes and repeat 5 times. The regenerated sample is placed in a fresh solution of the analyte (B4) for 30 minutes, and then the fluorescence intensity is collected. Repeat the above steps 5 times.

The fluorescence quenching was analyzed using the Stern-Volmer equations:

$$\mathbf{(I_0 - I)/I_0 = \boldsymbol{K}_{sv}[Q]} \qquad \text{Equation (1)}$$

where I_0 and I are the fluorescence intensity, in the absence and presence of crystal, respectively, $\boldsymbol{K}_{sv}$ is the Stern-Volmer quenching constant and [Q] is the concentration of the analyte. The quenching percentage was calculated using the equation as follows:
Fluorescence quenching % = $(1 - I/I_0) \times 100$ %
where I_0 is the initial fluorescence intensity in the absence of crystal, I is the fluorescence intensity in the presence of corresponding crystal.
The limit of detection concentration (LOD) was calculated according to the equation (2):

$$\mathbf{LOD = 3\delta/K_{sv}} \qquad \text{Equation (2)}$$

and δ is the standard deviation of the detection method.

The density functional theory (DFT) calculation

(i) The thermodynamic stability of homochiral and heterochiral cage of **1**

The heterochiral cage is constructed by replacing one S-BINAM moiety in the homochiral cage with R-BINAM. Then both the homochiral and heterochiral cages were optimized at PBE0/6-31G(d,p) level in Gaussian 16 package,[1] which are shown in Fig. 2. Subsequent frequency calculation at PBE0/6-31G(d,p) level helps us to obtain some parameters related to thermodynamic stability, such as the sum of electronic and thermal enthalpies (H), the sum of electronic and thermal free energies (G), total entropy (S) and their corresponding differences (ΔH, ΔG and ΔS) at 298.15 K, which are listed in Supplementary Table 2.

(ii) The intermolecular interactions between cages and organic solvent

Based on the structures selected from the crystals of **1-*R*/*S***, **1-*S*** and **1-*R***, we estimated various intermolecular interactions using DFT method. The intermolecular interactions energies were calculated considering basis-set superposition error (BSSE) correction at the PBE0-D3/6-G(d,p) level, which employs the Grimmers' dispersion correction scheme in order to give a better description of intermolecular weak interaction.[2]

(iii) The recognition of enantiomer ***R*(*S*)-B4** by **1-*S***

The general AMBER force field (GAFF) with the restricted electrostatic potential (RESP) charges was built for **1-S** and ***R*-B4** (***S*-B4**) molecules in GROMACS[3, 4]. Short-range electrostatics and short-range van der Waals (rvdW) were applied with a spherical cut-off of 1.2 nm. Also, Particle Mesh Ewald (PME) was used for long-range electrostatics and a steep integrator was adopted. Initial energy minimization was carried out and a cluster model was obtained with the same mole ratio of ***R*-**, ***S*-B4** and **1-*S*** in the experiment. The isothermal-isobaric ensemble (NPT) simulation was conducted in the V-rescale Berendsen thermostat and barostat for 10 ns at 300K and 1 Torr. The total energy evolves with the simulation time are presented in Supplementary Figure 31, which suggests the good balance of these two sytems. Then, a dimer model of ***R*-**, ***S*-B4** and **1-*S*** blend was extracted from the molecular dynamics simulated cluster model as shown in Supplementary Figure 32 and 33. Subsequently, a further geometrical optimization on the extracted dimer was carried out at PBE0-D3/6-31G(d,p) level, which is exhibited in Supplementary Figure 32(c) and 33(c). Based on the optimized structures of **1-*S*/*R*-B4** and **1-*S*/*S*-B4**, the counterpoised-corrected intermolecular interaction energies (E_{int}) and electronic couplings (EC_{ET}) for excitation energy transfer were evaluated, among which the theoretical method for the energy transfer referred to the formulation of Iozzi, Mennucci, Tomasi and Cammi performed in Gaussian 16[5].

All above DFT calculations were performed in Gaussian 16 packadge.

Supplementary Figures

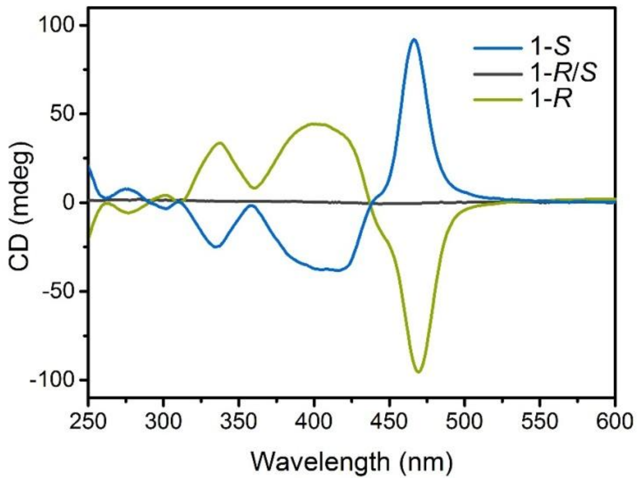

Supplementary Figure 2. CD spectra. CD spectra (KBr pellets) of **1-*S***, **1-*R*** and **1-*R*/*S*⊃mesitylene**.

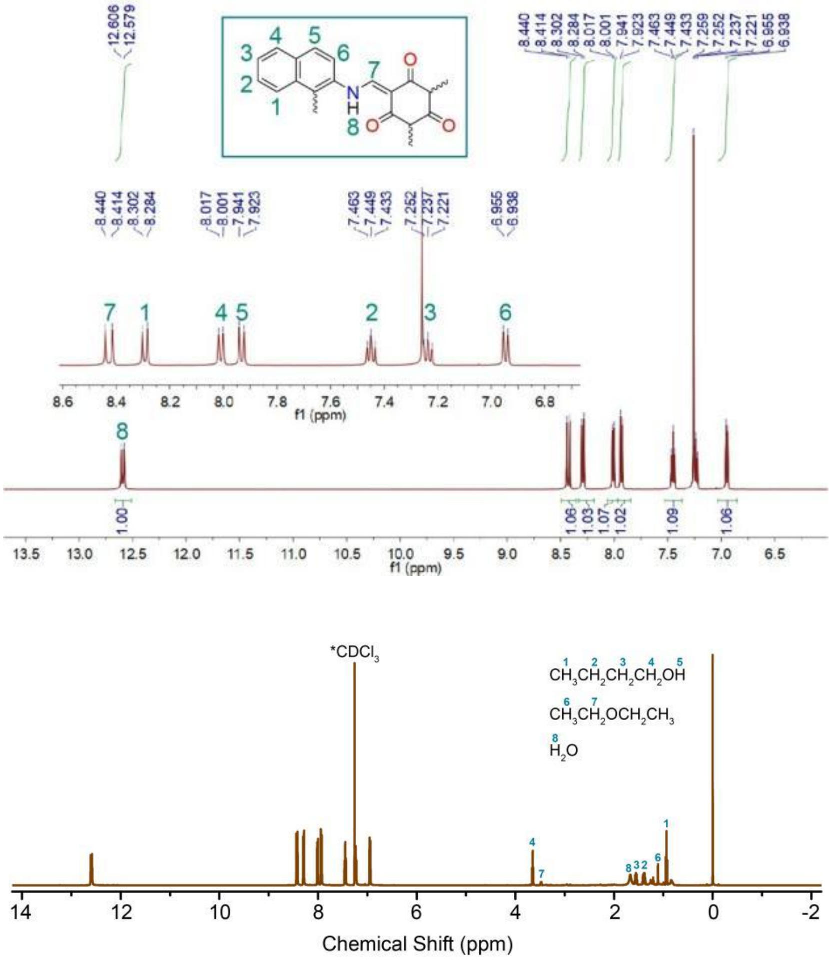

Supplementary Figure 3. ^{1}H NMR spectrum. ^{1}H NMR spectrum of **1-*S*** in $CDCl_3$ (500M at 25 °C). The top one is the enlarged spectrum and the bottom one is the full spectrum.

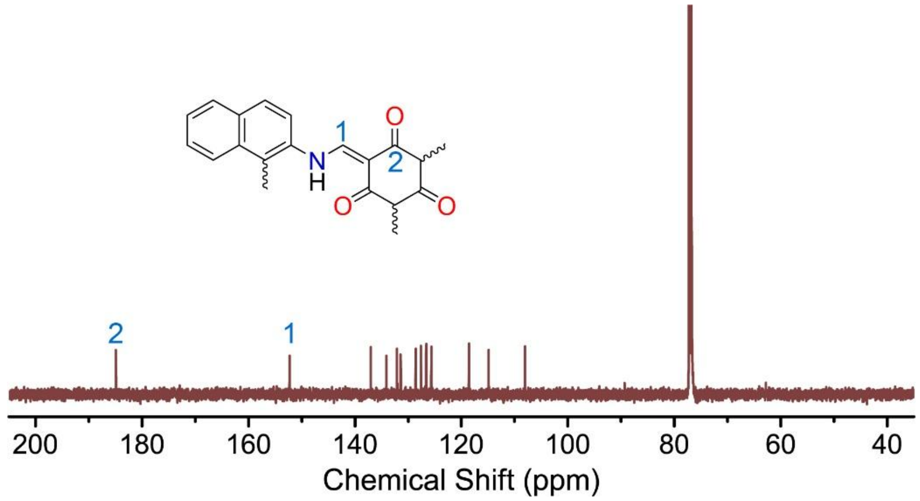

Supplementary Figure 4. ^{13}C NMR spectrum of **1-*S*** in $CDCl_3$ (500M at 25 °C).

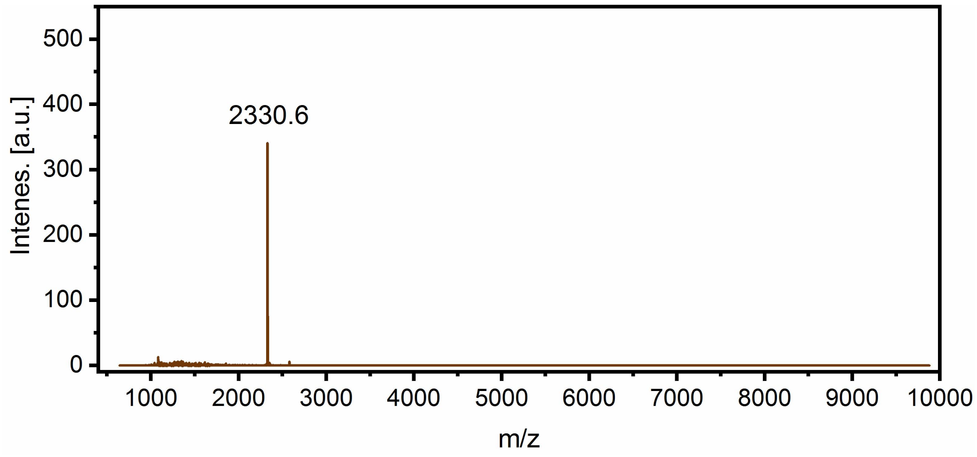

Supplementary Figure 5. Mass spectrum of 1-*S*. Mass spectrum of **1-*S*** and the characteristic peak at 2330.6 is marked.

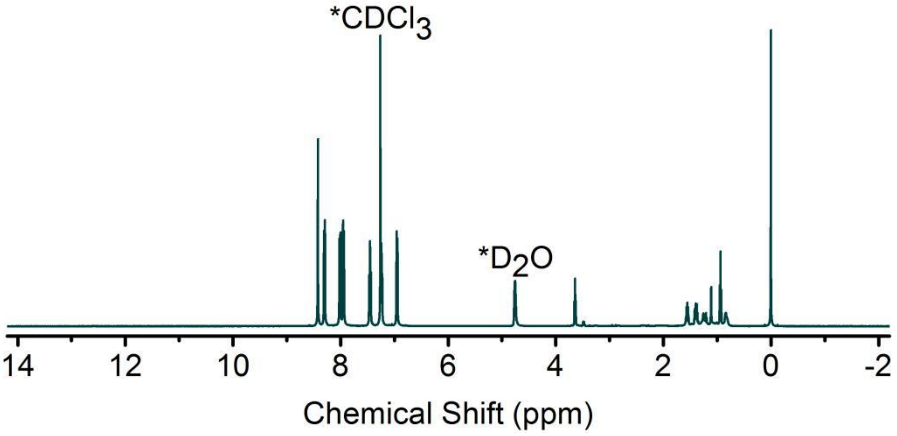

Supplementary Figure 6. ^{1}H NMR spectrum. ^{1}H NMR spectrum of **1-*S*** after addition of D_2O in $CDCl_3$ (500M at 25 °C).

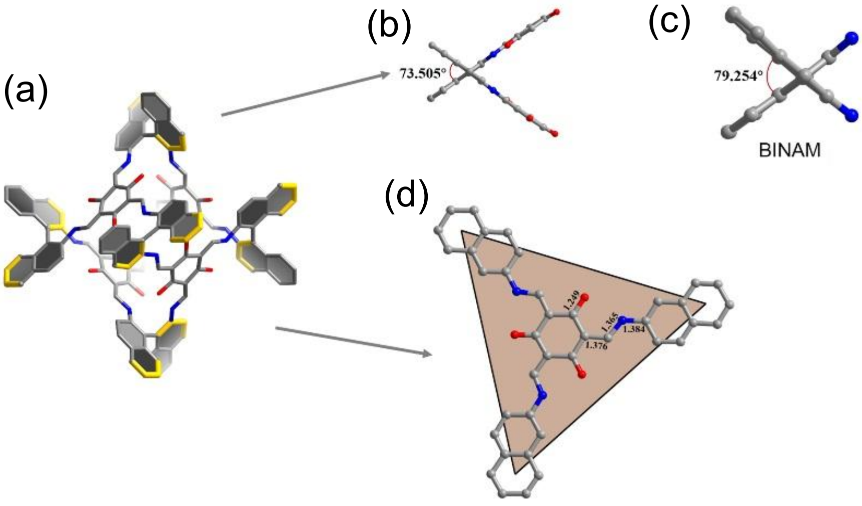

Supplementary Figure 7. Dihedral angle and bond length in the cage. (a) The structure of the cage. (b) and (c) The dihedral angle in the cage. (d) Bond length in the cage.

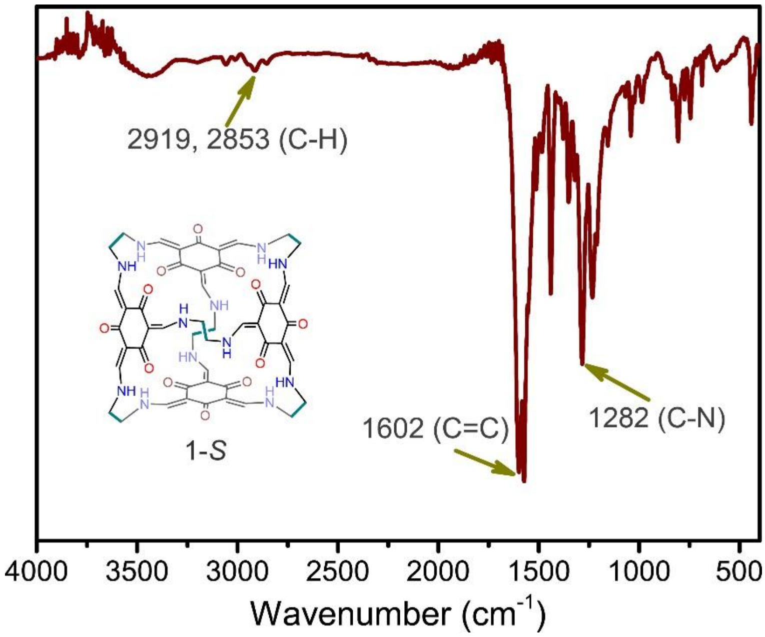

Supplementary Figure 8. FTIR spectrum. The FTIR spectrum of **1-*S*** via KBr pellets. The characteristic vibration peaks of C-H, C=C and C-N bongs are marked. The insert is the chemical structure of **1-*S***.

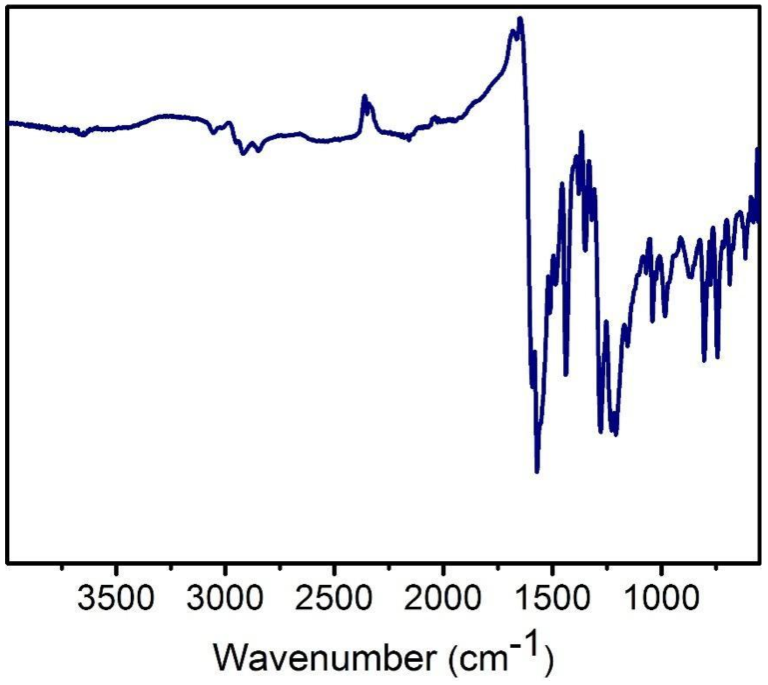

Supplementary Figure 9. IR spectrum of 1-*S*. The IR spectrum of **1-*S*** with air background.

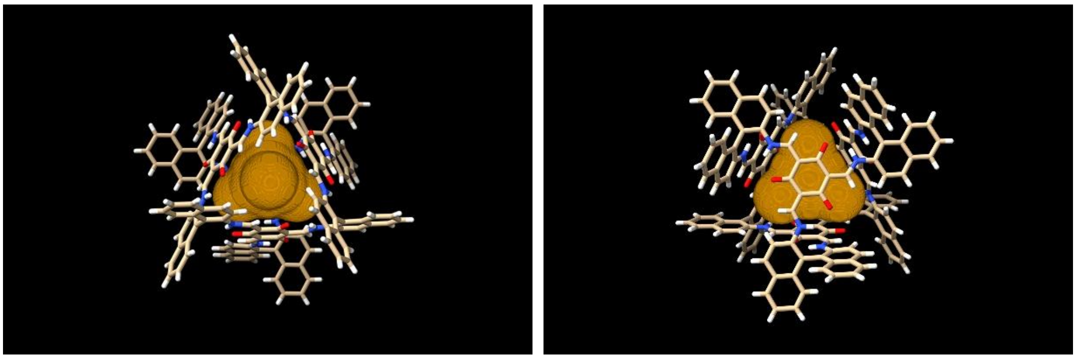

Supplementary Figure 10. The Schematic illustration of void spaces. VOIDOO-calculated void spaces (yellow mesh) within the homochiral cage.

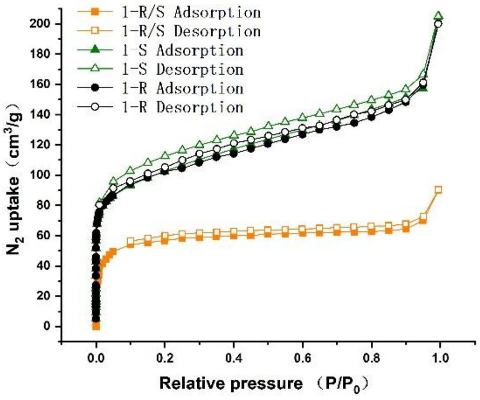

Supplementary Figure 11. N_2 adsorption isotherms. N_2 adsorption isotherms of activated **1-*S***, **1-*R*** and **1-*S*/*R*** at 1.0 bar under 77 K.

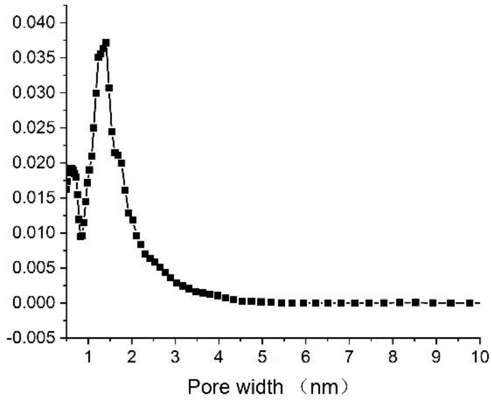

Supplementary Figure 12. Pore size distribution. Pore size distribution of **1-*S***.

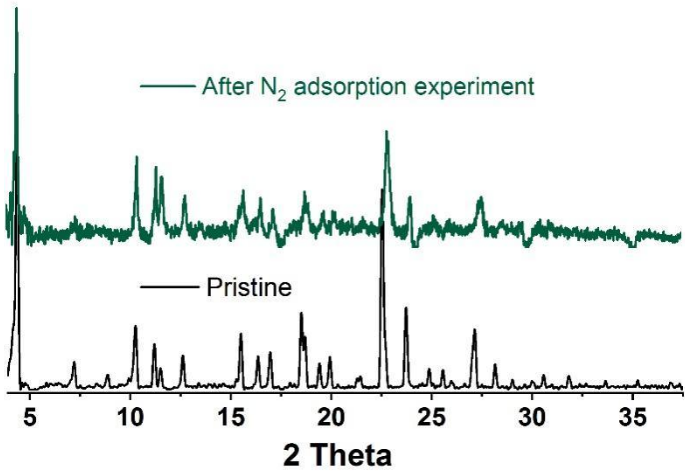

Supplementary Figure 13. PXRD patterns. The PXRD patterns of fresh **1-*S*** (black line) and **1-*S*** after N_2 adsorption experiment (green line).

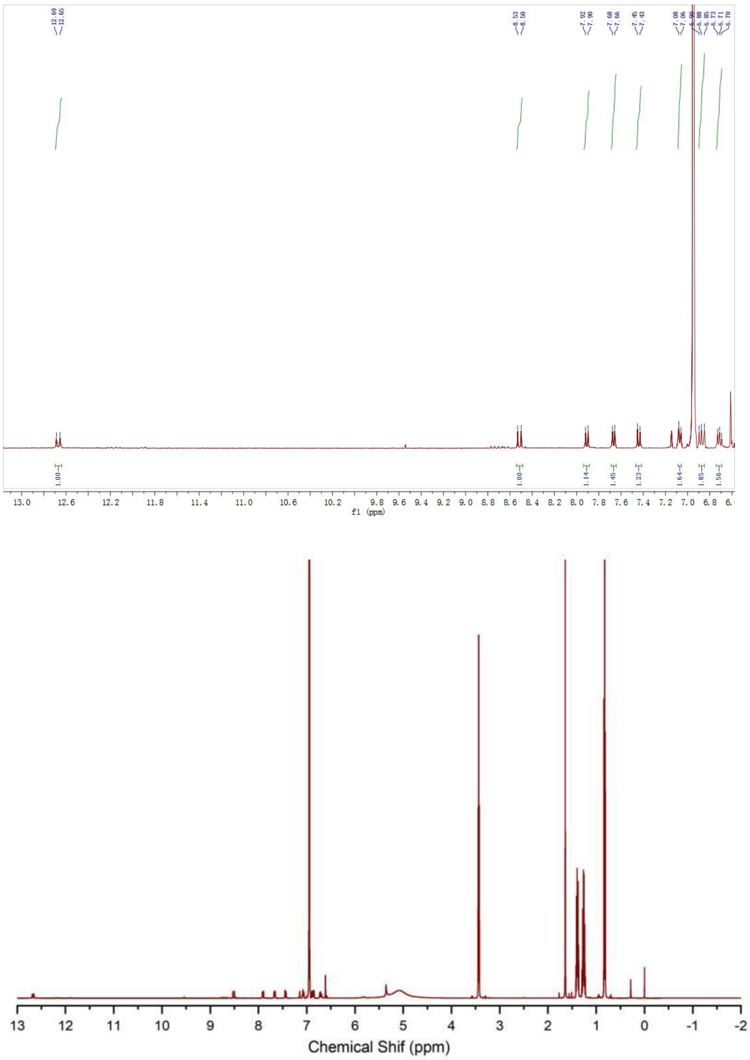

Supplementary Figure 14. The in situ ^{1}H NMR spectrum. The in situ ^{1}H NMR spectrum in benzene-d6 (500M at 25 °C). The top one is enlarged and the bottom is the full spectrum.

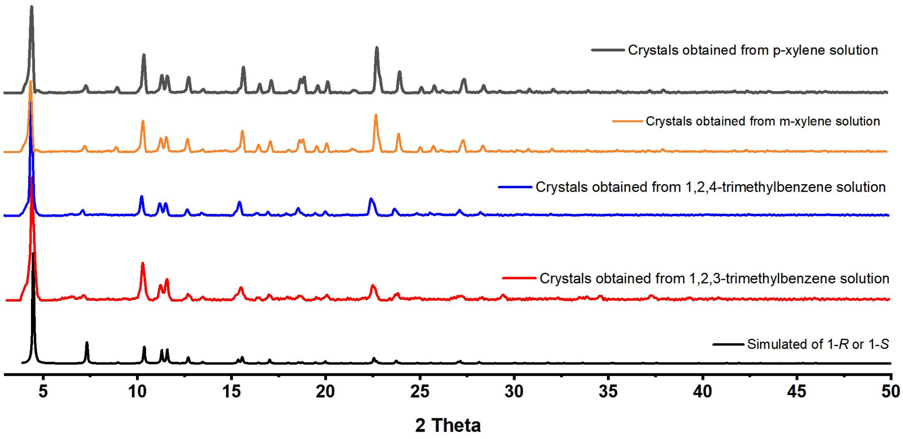

Supplementary Figure 15. PXRD patterns of the sample under different solvent. PXRD patterns of the synthesized crystals from p-xylene, m-xylene, 1,2,4-trimethylbenzene, 1,2,3-trimethylbenzene.

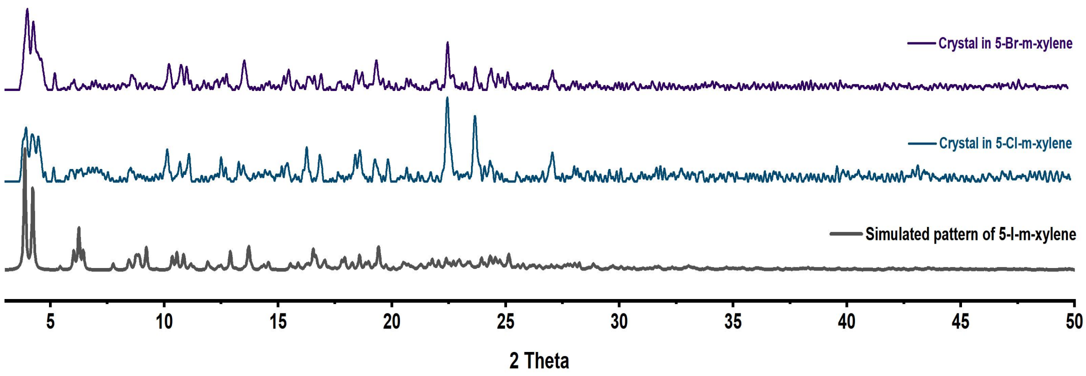

Supplementary Figure 16. PXRD patterns of the sample under different halogenated benzene solvent. PXRD patterns of the synthesized crystals 5-Cl-m-xylene, 5-Br-m-xylene and 5-I-m-xylene.

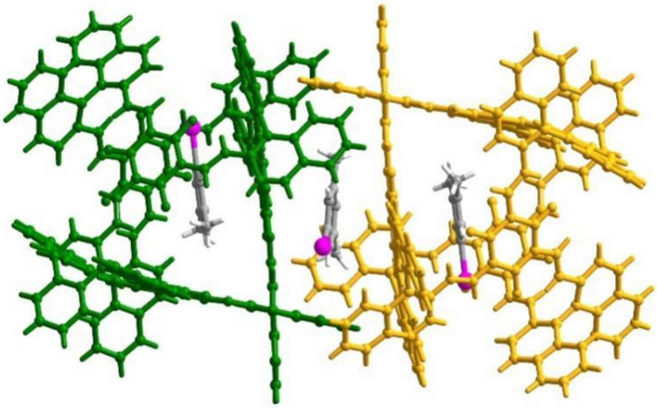

Supplementary Figure 17. The cage structures of 1-*R*/*S*⊃5-iodo-m-xylene. The green one represents the ***S*** cage and the yellow represnts ***R*** cage. The pink atom is iodine and the grey atom is carbon.

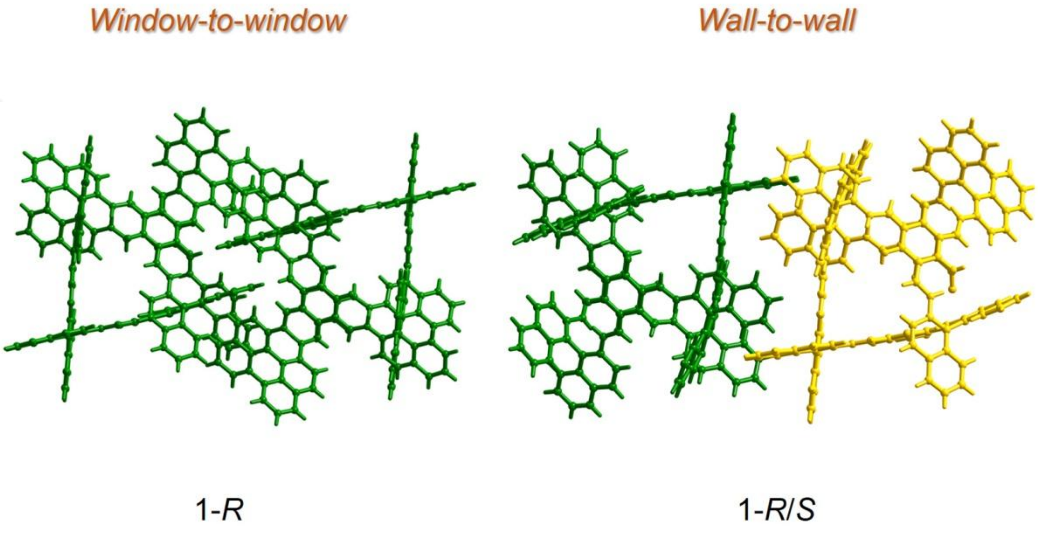

Supplementary Figure 18. The packing mode in the cages. The window-to-window packing in homochiral **1-*R*** (left) and the wall-to-wall packing in heterochiral packing of **1-*R*/*S***.

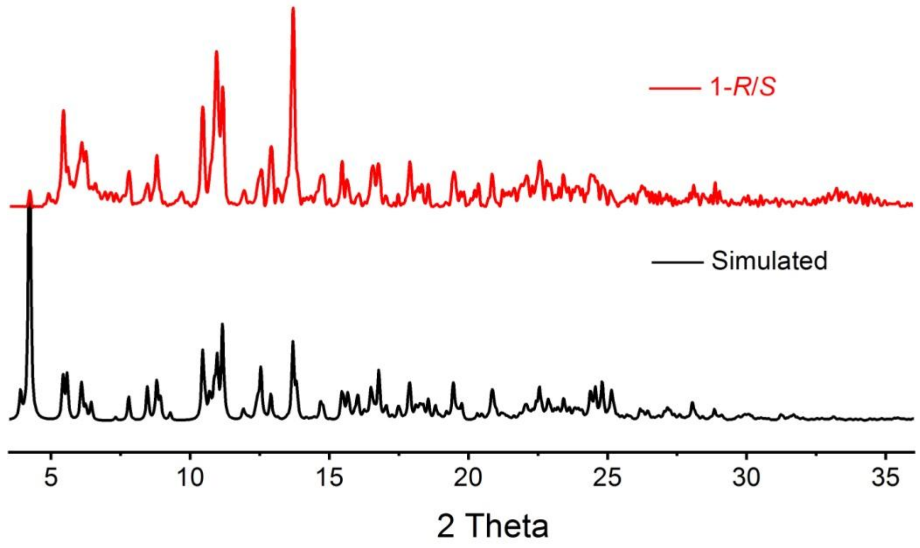

Supplementary Figure 19. PXRD patterns of 1-*R*/*S*⊃mesitylene. The synthesized (red) and simulated (black) PXRD patterns of **1-*R*/*S*⊃mesitylene**.

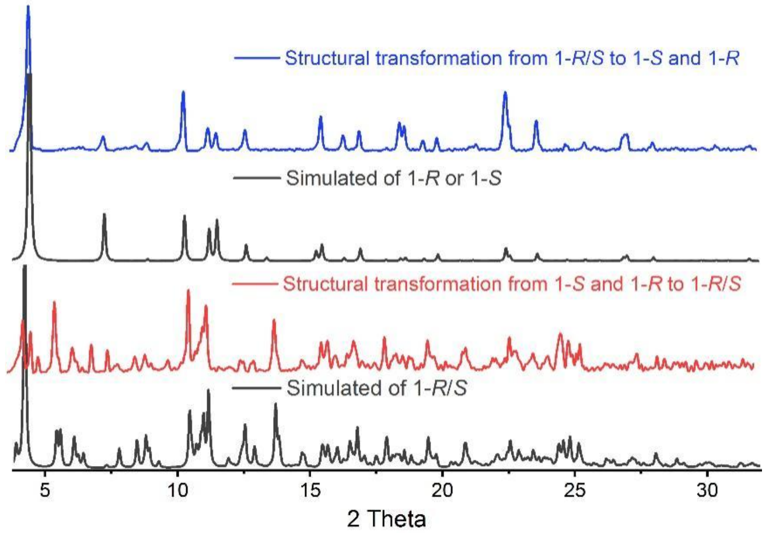

Supplementary Figure 20. PXRD patterns of cages after structural transformation. Structural transformation from **1-*R*/*S*** to **1-*R*** and **1-*S*** (blue line), simulated one of **1-*R*** or **1-*S*** (black line), structural transformation from **1-*R*** and **1-*S*** to **1-*R*/*S*** (red line) and simulated one of **1-*R*/*S***.

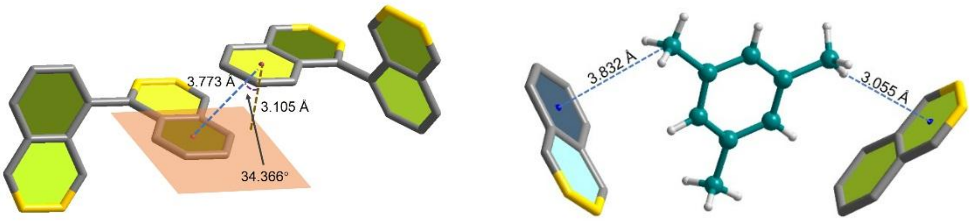

Supplementary Figure 21. Schematic illustration of supramolecular interactions. The supramolecular interactions between octahedral in **1-*R*** (left) and **1-*R*/*S*⊃mesitylene** (right).

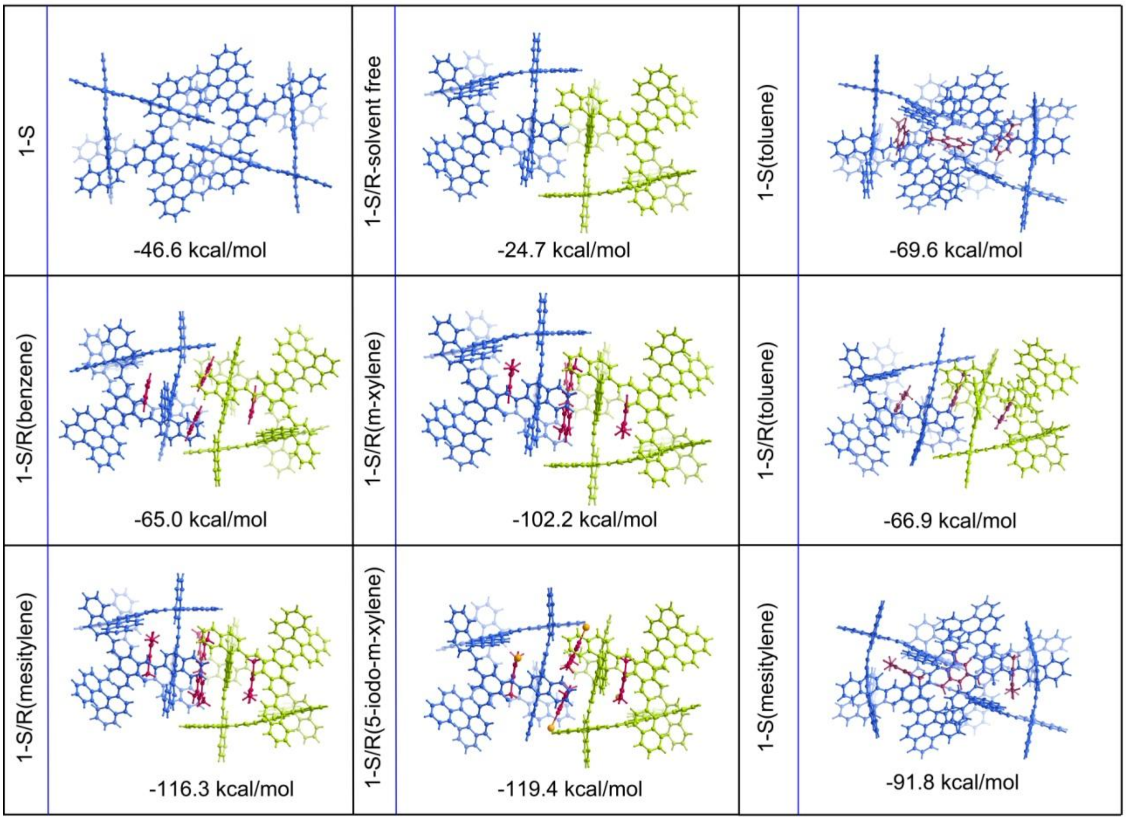

Supplementary Figure 22. DFT calculated interaction energies. DFT calculated interaction energies for stimulated structures of homochiral and racemic structures with or without solvent.

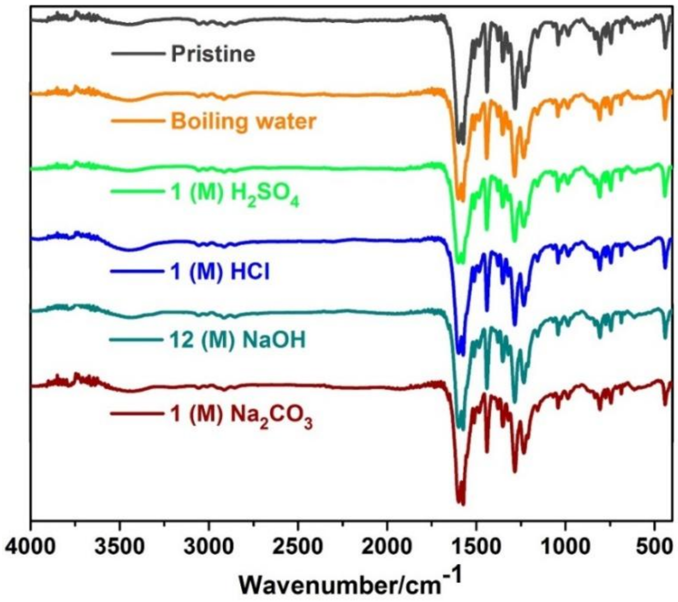

Supplementary Figure 23. FTIR spectrum (KBr pellets) of 1-*S* after soaking under different conditions. FTIR spectrum of the pristine **1-*S*** (black), under boiling water (orange), 1M H_2SO_4 (green), 1M HCl (blue), 12M NaOH (dark blue), 1M Na_2CO_3 (dark red).

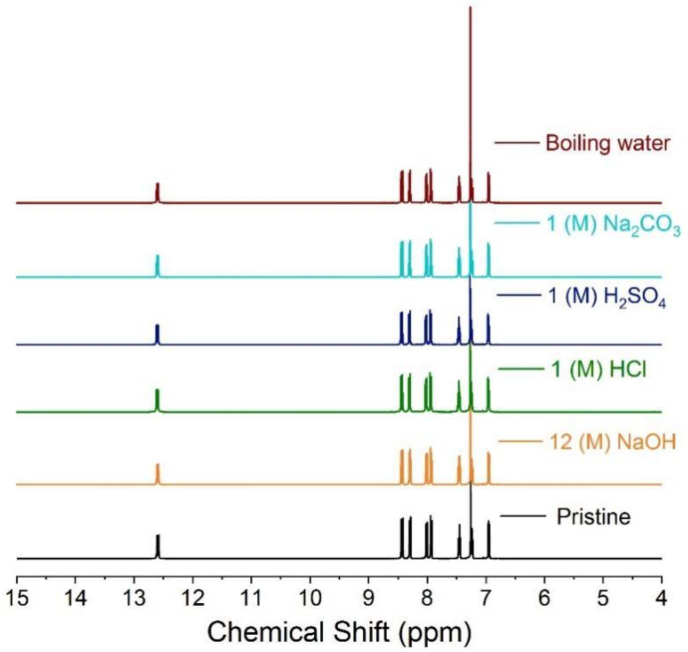

Supplementary Figure 24. ^{1}H NMR spectra in $CDCl_3$ of **1-*S*** after soaking under different conditions (500M at 25 °C). ^{1}H NMR spectra of the pristine **1-*S*** (black), under boiling water (red), 1M Na_2CO_3 (bright blue).1M H_2SO_4 (dark blue), 1M HCl (green) and 12M NaOH (orange).

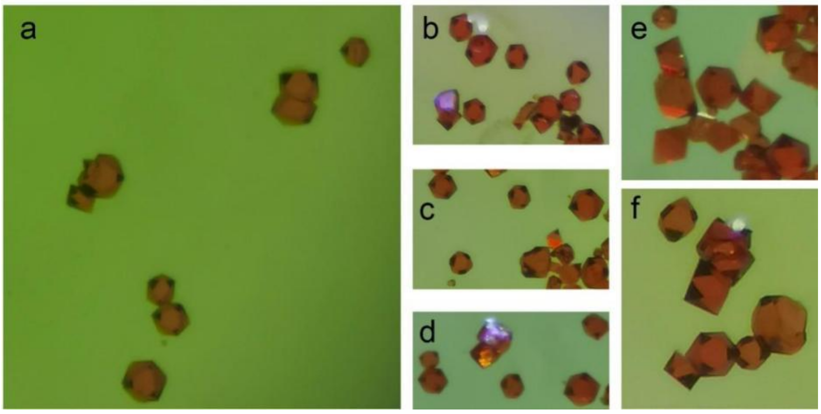

Supplementary Figure 25. The photos of 1-*S* crystals. The photos of **1-*S*** before and after immersed in harsh conditions for 7 day. (a) before the treatment, (b) boiling water, (c) 1M Na_2CO_3, (d) 1M H_2SO_4, (e) 1M HCl, and (f) 12M NaOH.

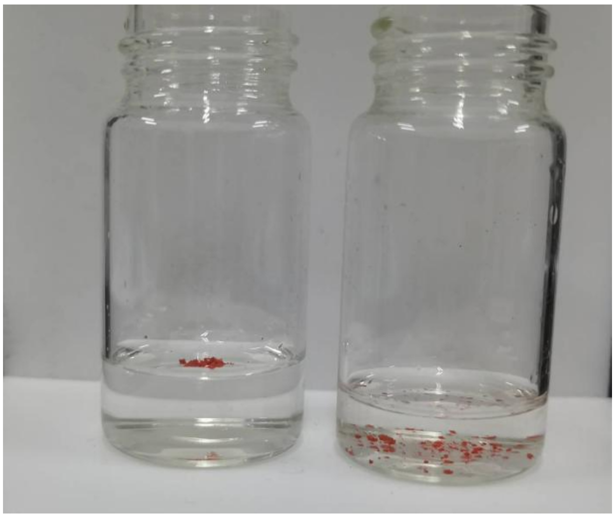

Supplementary Figure 26. The photos of 1-*S* crystals under different condition. The photo of **1-*S*** sample in 1M H_2SO_4 aqueous solution (left) and 1M H_2SO_4/MeOH/H_2O solution (right).

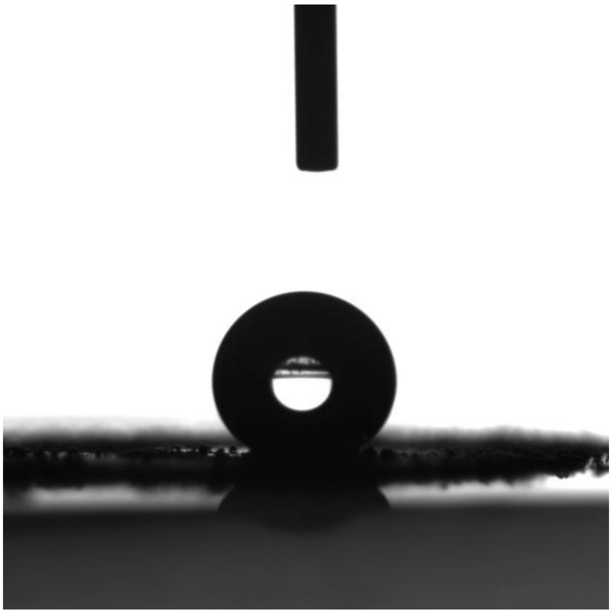

Supplementary Figure 27. The water contact angle. The water contact angle of 145.7° for **1-*S***.

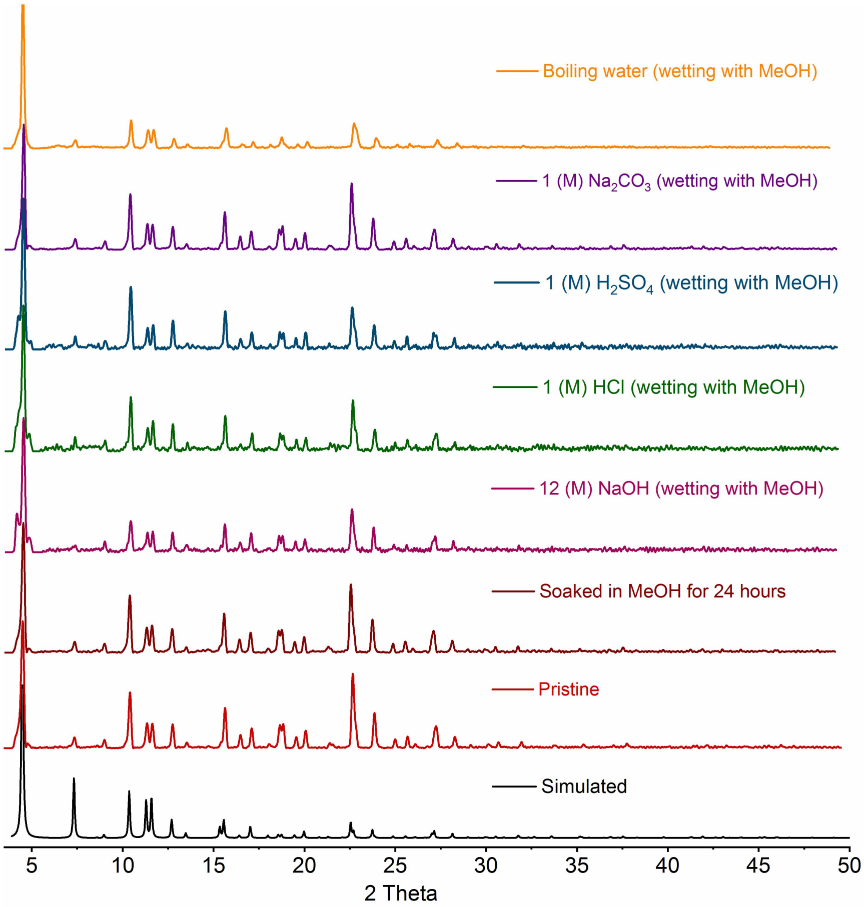

Supplementary Figure 28. PXRD patterns of 1-*S* under different harsh conditions after wetting with MeOH. The simulated (black), the pristine **1-*S*** (red), in MeOH (dark red), 12M NaOH (pink), 1M HCl (green), 1M H_2SO_4 (dark blue), 1M Na_2CO_3 (purple) and boiling water (orange).

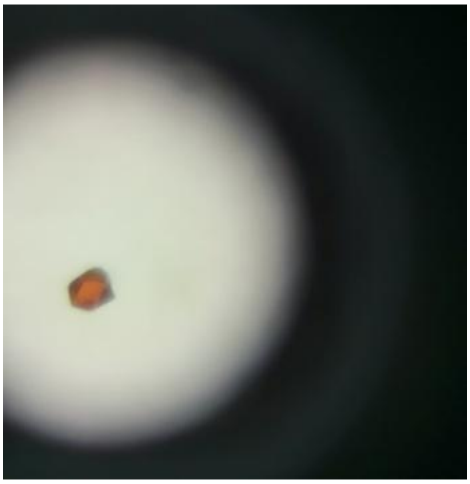

Supplementary Figure 29. The photo of a 1-*S* crystal. The photo of a 1-*S* crystal after immersed in 1M H_2SO_4/MeOH/H_2O for 7 d.

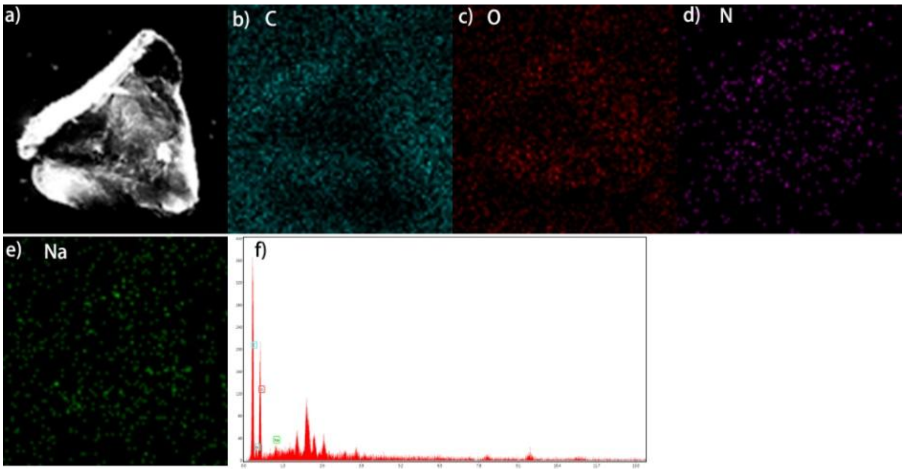

Supplementary Figure 30. The SEM images of the cross-section of a crystal after soaked in12M NaOH/MeOH/H_2O for 7d. (a) The SEM images. (b-e) element mapping of C, O, N, Na. (f) The EDX spectrum.

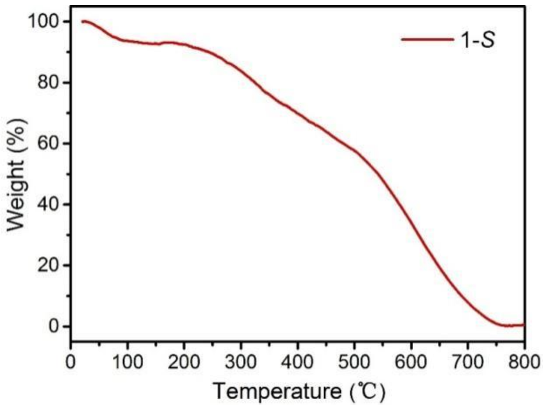

Supplementary Figure 31. TGA curve. TGA curve of **1-*S*** in the range of 20-800°C under N_2 atmosphere.

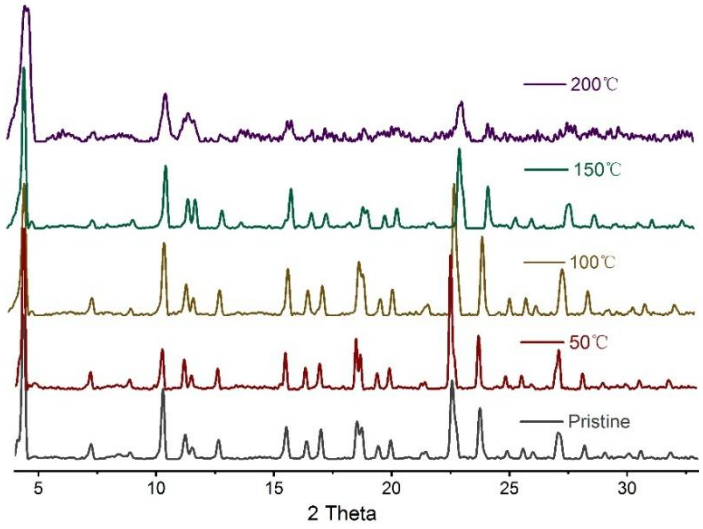

Supplementary Figure 32. PXRD patterns at of 1-*S* at different temperature. PXRD patterns of **1-*S*** after heating for 15 minutes at 200 °C (purple), 150 °C (green), 100 °C (purple), 50 °C (red).

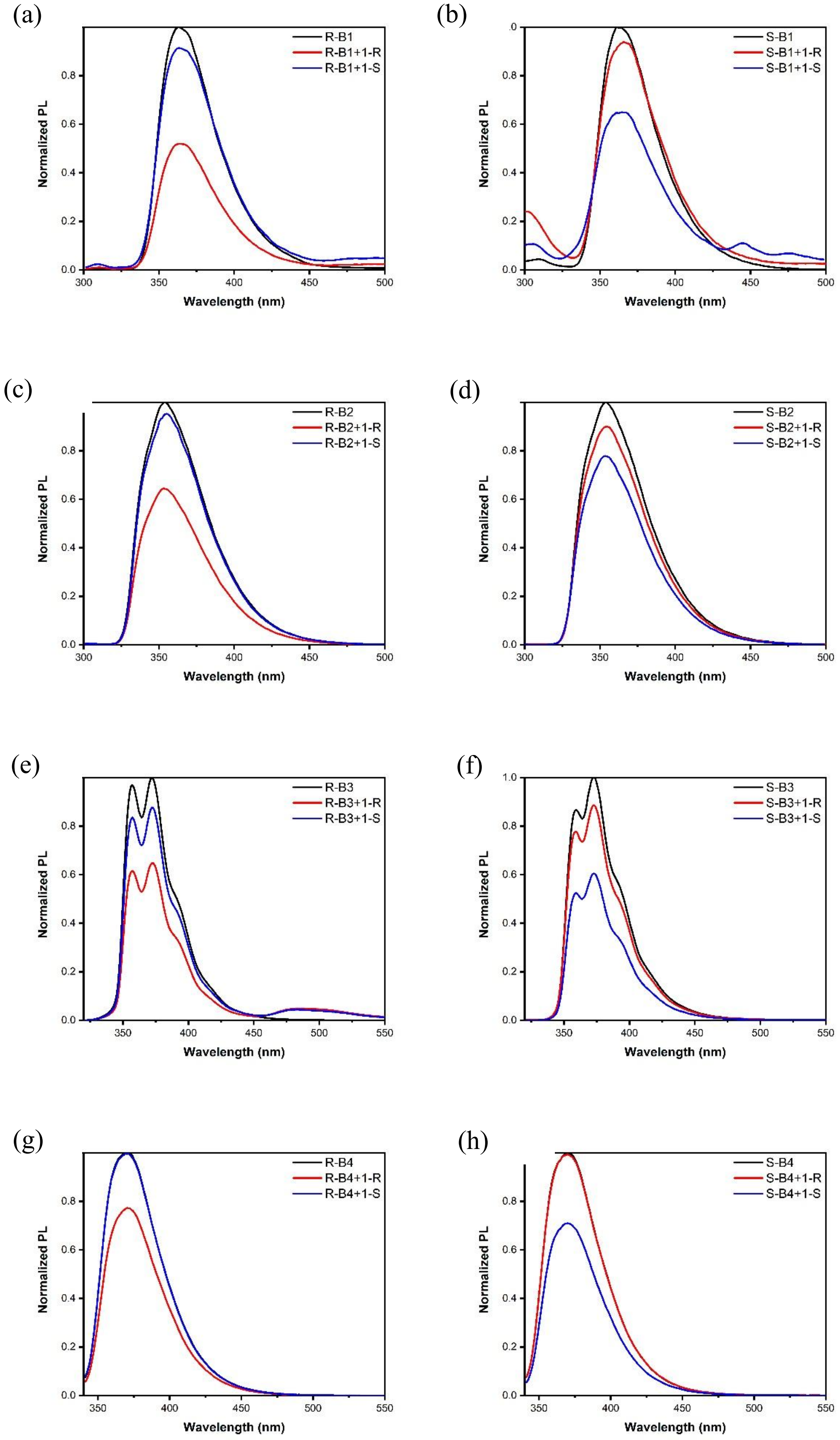
(a)
R-B1
R-B1+1-R
R-B1+1-S
Normalized PL
Wavelength (nm)
(b)
S-B1
S-B1+1-R
S-B1+1-S
Normalized PL
Wavelength (nm)
(c)
R-B2
R-B2+1-R
R-B2+1-S
Normalized PL
Wavelength (nm)
(d)
S-B2
S-B2+1-R
S-B2+1-S
Normalized PL
Wavelength (nm)
(e)
R-B3
R-B3+1-R
R-B3+1-S
Normalized PL
Wavelength (nm)
(f)
S-B3
S-B3+1-R
S-B3+1-S
Normalized PL
Wavelength (nm)
(g)
R-B4
R-B4+1-R
R-B4+1-S
Normalized PL
Wavelength (nm)
(h)
S-B4
S-B4+1-R
S-B4+1-S
Normalized PL
Wavelength (nm)

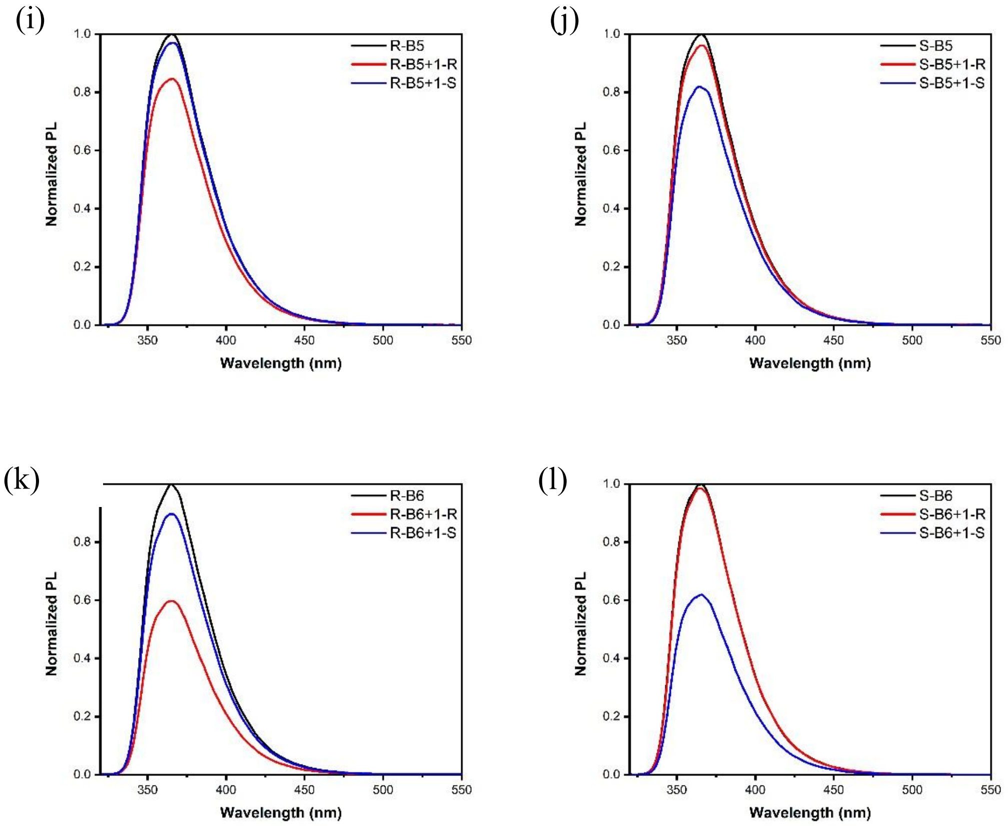

Supplementary Figure 33. Fluorescence spectra of 1-*S* and 1-*R* under different atropisomerically biaryl molecules in EtOH and H_2O 1:1 (v/v) under the excitation wavelength of 370 nm. (a) *R*-**B1**, (b)*S*-**B1**, (c)*R*-**B2**,(d) *S*-**B2**, (e)*R*-**B3**,(f)*S*-**B3**, (g)*R*-**B4**,(h) *S*-**B4**, (i)*R*-**B5**,(j) *S*-**B5** and (k)*R*-**B6**,(l) *S*-**B6**.

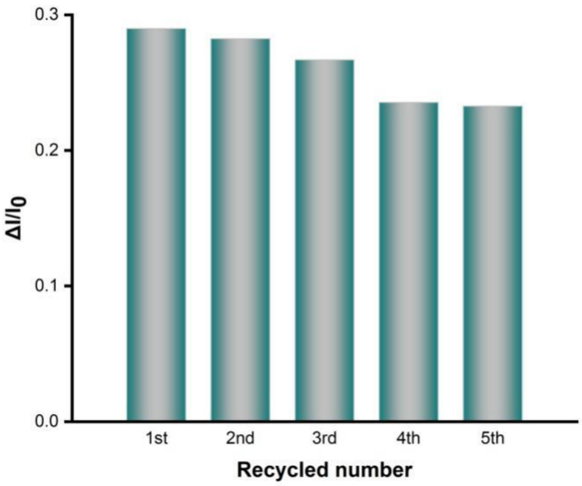

Supplementary Figure 34. Ratio of luminescent intensity. Recyclability experiment of **1-*S*** upon titration of *S*-B4 in EtOH and H_2O 1:1 (v/v) under the excitation wavelength of 370 nm.

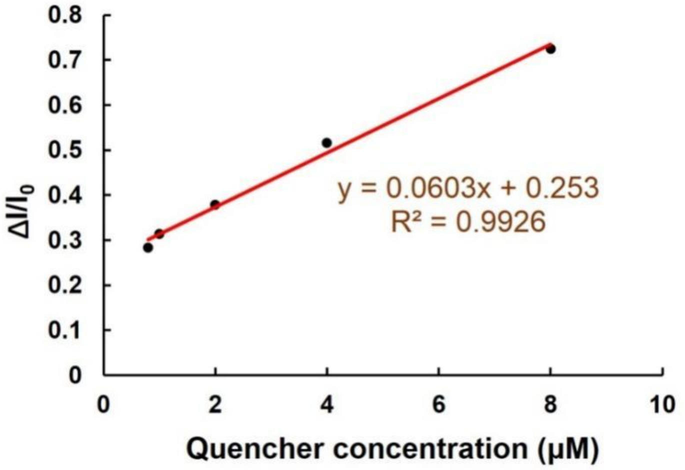

Supplementary Figure 35. The Stern−Völmer plot. The Stern−Völmer plot of **1-*S*** and *S*-B4 in EtOH and H_2O 1:1 (v/v).

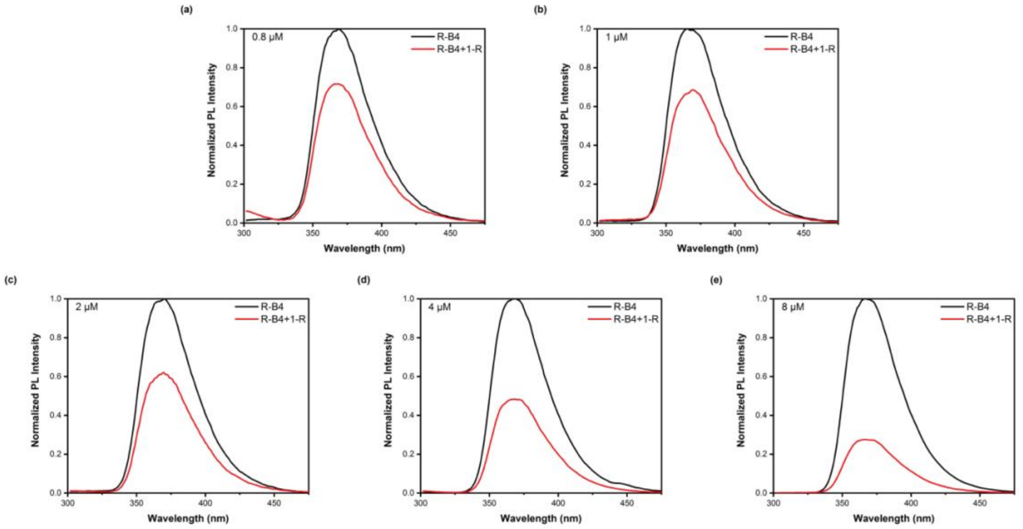

Supplementary Figure 36. Fluorescence spectra of *S*-B4 with different concentration of R-B4 (EtOH/H_2O solvent) under the excitation wavelength of 370 nm. (a) 0.8 μM, (b) 1.0 μM, (c) 2.0 μM, (d) 4.0 μM and (e) 8.0 μM.

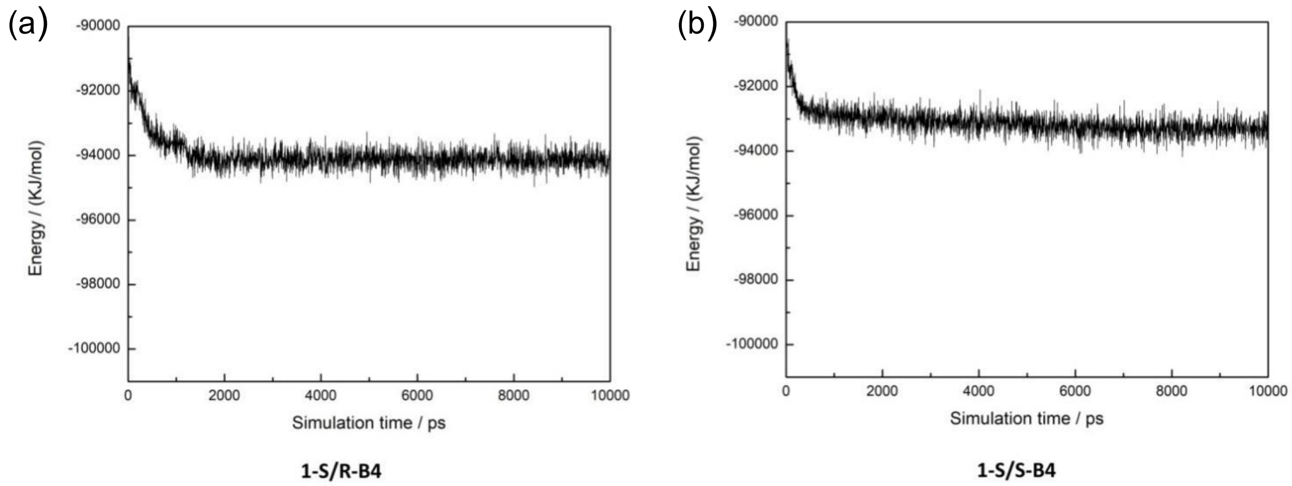

Supplementary Figure 37. The plots of energy versus simulation time in NPT (Normal Pressure and Temperature) process. (a) **1-*S*/*R*-B4** blend. (b) **1-*S*/*S*-B4** blend.

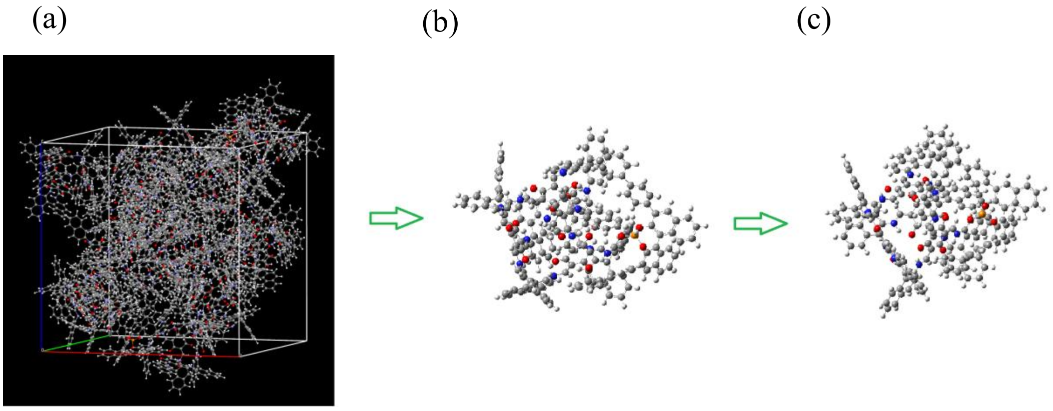

Supplementary Figure 38. The cluster model of 1-*S*/*R*-B4 blend. (a) in the box after molecular dynamics simulation for **1-*S*** and ***R*-B4**, the extracted dimer model (b) in **1-*S*/*R*-B4** blend, and the optimized dimer (c) at PBE0-D3/6-31G(d,p) level based on the extracted model.

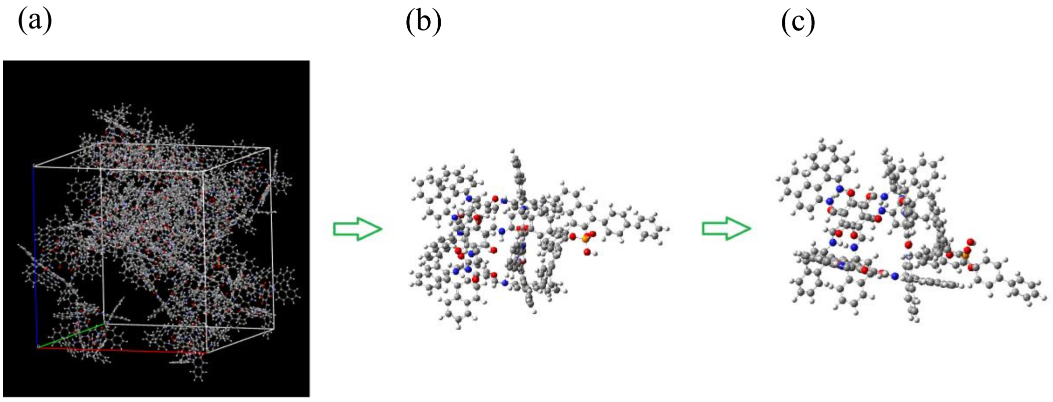

Supplementary Figure 39. The cluster model of 1-*S*/*S*-B4 blend. (a) in the box after molecular dynamics simulation for **1-*S*** and ***S*-B4**, the extracted dimer model (b) in **1-*S*/*S*-B4** blend, and the optimized dimer (c) at PBE0-D3/6-31G(d,p) level based on the extracted model.

Supplementary Tables

Supplementary Table 1. The Crystallographic data for **1-*R***, **1-*S*** and **1-*R*/*S*** guest.

Compound	**1-*R***	**1-*S***	**1-*R*/*S*⊃mesitylene**	**1-*R*/*S*⊃5-iodo-m-xylene**
Empirical formula	$C_{156}H_{96}N_{12}O_{12}$	$C_{156}H_{96}N_{12}O_{12}$	$C_{192}H_{144}N_{12}O_{12}$	$C_{188}H_{132}N_{12}O_{12}$
Formula weight	2330.45	2330.44	2811.18	3258.65
Temperature/K	100.0	100.0	173.0	173.0
Crystal system	Cubic	Cubic	Triclinic	Triclinic
Space group	$F4_132$	$F4_132$	*P*-1	*P*-1
a/Å	33.4113 (5)	33.4734(4)	16.7006(19)	16.7558(7)
b/Å	33.4113 (5)	33.4734(4)	21.837(2)	21.8576(9)
c/Å	33.4113 (5)	33.4734(4)	23.238(3)	23.3366(10)
α/°	90	90	80.823(5)	81.288(2)
β/°	90	90	78.270(6)	78.906(2)
γ/°	90	90	74.176(5)	73.899(2)
Volume/Å^3	37297.5 (17)	37505.9(13)	7934.4(15)	8014.4(6)
Z	8	8	2	2
ρ_{calc}g/cm^3	0.830	0.825	1.177	1.350
μ/mm^{-1}	0.049	0.050	0.582	6.626
F(000)	9696.0	9696.0	2952.0	3304.0
2θ	7.328 to 127.54	7.444 to 144.6	5.58 to 80.336	5.436 to 80.358
Reflections collected	125750	89535	63517	47837
Independent reflections	3588	3229	9465	9776
GOOF	1.080	1.078	1.018	1.019
R_1 (I>2σ (I))	0.0922	0.1402	0.0900	0.1326
wR_2 (I>2σ (I))	0.2652	0.3325	0.2614	0.3268

Supplementary Table 2. The behaviors of supramolecular chiral self-sorting under different solvent.

Item	Solvent	Chiral self-sorting on cage formation		Chiral self-sorting on supramolecular level	
		Homochirality	Heterochirality	Homochirality	Heterochirality
1	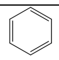	√		√	
2	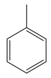	√		√	
3	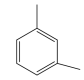	√		√	
4	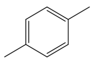	√		√	
5	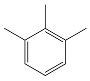	√		√	
6	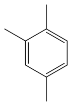	√		√	
7	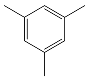	√			√
8	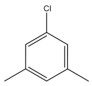	√			√
9	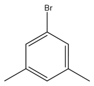	√			√
10	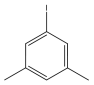	√			√

Supplementary Table 3 The calculated sum of electronic and thermal enthalpies (H), sum of electronic and thermal free energies (G), total entropy (S) and their corresponding differences (ΔH, ΔG and ΔS) at PBE0/6-31G(d,p) level.

	H (a.u.)	ΔH (kJ·mol^{-1})	G (a.u.)	ΔG (kJ·mol^{-1})	S		ΔS	
					(J·K^{-1}·mol^{-1})	(kJ·mol^{-1})	(J·K^{-1}·mol^{-1})	(kJ·mol^{-1})
Homochiral cage	-7551.81		-7552.13		2820.09	840.81		
Heterochiral cage 1	-7551.77	105.35	-7552.09	102.25	2830.50	843.91	10.41	3.10
Heterochiral cage 2-1	-7551.73	210.72	-7552.05	209.47	2815.97	839.58	-4.13	-1.23
Heterochiral cage 2-2	-7551.71	273.18	-7552.03	277.27	2806.39	836.72	-13.70	-4.08
Heterochiral cage 3-1	-7551.69	312.33	-7552.01	316.41	2806.42	836.73	-13.67	-4.07
Heterochiral cage 3-2	-7551.69	311.93	-7552.01	316.44	2804.98	836.30	-15.11	-4.50

Supplementary Table 4. Elemental analysis of **1-*S***.

	C	H	N
Calcd	80.41	4.12	7.22
Pristine	80.24	4.30	6.98
Boiling water	79.82	4.51	7.02
1 (M) Na_2CO_3	80.31	4.22	7.16
1 (M) HCl	79.96	4.47	6.88
1 (M) H_2SO_4	79.63	4.60	7.11
12 (M) NaOH	79.67	4.75	7.20

Supplementary Table 5 Summary of chemical stability of organic cages.

Name	Tolerance of structure			Supplementary Reference
	acidity	alkalinity	water	
Cage **1**	1 (M) HCl/H_2SO_4	12 (M) NaOH	stable	**This work**
CC3	unstable	unstable	stable	6
CC3-mix, CC3-trans	humid vapor and aqueous SO_2	/	/	7
FT-RCC3	pH=1.7	pH=12.3	stable	8
Cage **1**	pH=3	pH=11	/	9
Cage **2**	pH=-1.9	pH=15.2	/	9
TpBDA	0.5 (M) HCl	1 (M) NaOH	stable	10
TpPNDA	0.5 (M) HCl	1 (M) NaOH	stable	10
NC1	/	/	stable	11
TpOMe-CDA	0.1 (M) HCl/H_2SO_4	12 (M) NaOH	stable	12
Cage **1**	unstable	unstable	/	13
Cage **3**	1 (M) HCl<br>10 (M) HCl 100°C	1 (M) NaOH	/	13

Supplementary Table 6. The Crystallographic data for **1-*S*** after soaked in 1M H_2SO_4/MeOH/H_2O for 7 d.

Compound	**1-*S***
Empirical formula	$C_{156}H_{96}N_{12}O_{12}$
Formula weight	2330.44
Temperature/K	100.0
Crystal system	Cubic
Space group	$F4_132$
a/Å	33.4206(5)
b/Å	33.4206(5)
c/Å	33.4206(5)
α/°	90
β/°	90
γ/°	90
Volume/Å^3	37328.7(17)
Z	8
ρ_{calc}g/cm^3	0.829
μ/mm^{-1}	0.050
F(000)	9696.0
Radiation	synchrotron(λ=0.6888)
Reflections collected	111873
Independent reflections	2981
GOOF	1.079
R_1 (I>2σ (I))	0.1281
wR_2 (I>2σ (I))	0.3365

Supplementary Table 7 The calculated intermolecular interaction energies E_{int} (kcal/mol) and electronic couplings EC_{ET} (meV) for excitation energy transfer between their first singlet excited state (S_1) at the optimized **1-*S*/*R*-B4** and **1-*S*/*S*-B4** dimers.

	E_{int}	EC_{ET}
**1-*S*/*R*-B4**	-41.18	0.62
**1-*S*/*S*-B4**	-37.74	2.57

Supplementary References

[1] Frisch MJ, *et al.* Fox Gaussian 16, Revision C.01, Gaussian Inc.: Wallingford CT (2019).

[2] Grimme S, Antony J, Ehrlich S. Krieg H. A Consistent and Accurate ab initio Parametrization of Density Functional Dispersion Correction, (DFT-D) for the 94 Elements H-Pu. *J. Chem. Phys.* **132**, 154104 (2010).

[3] Bayly CI, Cieplak P, Cornell W, Kollman, PA. A Well-behaved Electrostatic Potential Based Method Using Charge Restraints for Deriving Atomic Charges-the Rest Model *J. Phys. Chem.* **97**, 10269-10280 (1993).

[4] Fox T, Kollman PA, Application of the RESP Methodology in the Parametrization of Organic Solvents. *J. Phys. Chem. B* **102**, 8070-8079 (1998).

[5] Iozzi MF, Mennucci B, Tomasi J, Cammi R, Excitation Energy Transfer (EET) between Molecules in Condensed Matter: A Novel Application of the Polarizable Continuum M odel (PCM) *J. Chem. Phys.* **120**, 7029-7040 (2004).

[6] Hasell T, Schmidtmann M, Stone CA, Smith MW, Cooper AI. Reversible Water Uptake by a Stable Imine-based Porous Organic Cage *Chem. Commun.* **48**, 4689-4691 (2012).

[7] Zhu G, Hoffman CD, Liu Y, Bhattacharyya S, Tumuluri U, Jue ML, Wu Z, Sholl DS, Nair S, Jones CW, Lively RP. Engineering Porous Organic Cage Crystals with Increased Acid Gas Resistance. *Chem-Eur J.* **22**, 10743-10747 (2016).

[8] Liu M*, et al.* Acid- and Base-Stable Porous Organic Cages: Shape Persistence and pH Stability via Post-synthetic "Tying" of a Flexible Amine Cage. *J. Am. Chem. Soc.* **136**, 7583-7586 (2014).

[9] Alexandre PE, Zhang WS, Rominger F, Elbert SM, Schrçder RR, Mastalerz M. A Robust Porous Quinoline Cage: Transformation of a [4+6] Salicylimine Cage by Povarov Cyclization. *Angew. Chem. Int. Ed.* **59**, 19675–19679 (2020).

[10] Bera S*, et al.* Odd–Even Alternation in Tautomeric Porous Organic Cages with Exceptional Chemical Stability. *Angew. Chem. Int. Ed.* **56**, 2123-2126 (2017).

[11] Zhang L, Xiang L, Hang C, Liu W, Huang W, Pan Y. From Discrete Molecular Cages to a Network of Cages Exhibiting Enhanced CO2 Adsorption Capacity. *Angew. Chem. Int. Ed.* **2017**, *56*, 7787-7791.

[12] Bera S*, et al.* Porosity Switching in Polymorphic Porous Organic Cages with Exceptional Chemical Stability. *Angew. Chem. Int. Ed.* **58**, 4243-4247 (2019).

[13] Hu XY, Zhang WS, Rominger F, Wacker I, RR Schro¨derbc, Mastalerz M. Transforming a Chemically Labile [2+3] Imine Cage into a Robust Carbamate Cage. *Chem. Commun*. **53**, 8616-8619 (2017).
